# Ethoxy acetalated dextran-based nanocarriers accomplish efficient inhibition of leukotriene formation by a novel FLAP antagonist in human leukocytes and blood

**DOI:** 10.1007/s00018-021-04039-7

**Published:** 2021-12-31

**Authors:** Christian Kretzer, Blerina Shkodra, Paul Klemm, Paul M. Jordan, Daniel Schröder, Gizem Cinar, Antje Vollrath, Stephanie Schubert, Ivo Nischang, Stephanie Hoeppener, Steffi Stumpf, Erden Banoglu, Frederike Gladigau, Rossella Bilancia, Antonietta Rossi, Christian Eggeling, Ute Neugebauer, Ulrich S. Schubert, Oliver Werz

**Affiliations:** 1 Department of Pharmaceutical/Medicinal Chemistry, Institute of Pharmacy, Friedrich Schiller University Jena, Philosophenweg 14, 07743 Jena, Germany; 2 Laboratory of Organic and Macromolecular Chemistry (IOMC), Friedrich Schiller University Jena, Humboldtstraße 10, 07743 Jena, Germany; 3 Jena Center for Soft Matter (JCSM), Friedrich Schiller University Jena, Philosophenweg 7, 07743 Jena, Germany; 4 Institute of Applied Optics and Biophysics, Friedrich Schiller University Jena, Max-Wien Platz 1, 07743 Jena, Germany; 5 Department of Pharmaceutical Technology and Biopharmacy, Institute of Pharmacy, Friedrich Schiller University Jena, Lessingstraße 8, 07743 Jena, Germany; 6 Department of Pharmaceutical Chemistry, Faculty of Pharmacy, Gazi University, Etiler, Yenimahalle, Ankara, 06330 Turkey; 7 Leibniz Institute of Photonic Technology, Albert-Einstein-Straße 9, 07745 Jena, Germany; 8 Center for Sepsis Control and Care, Jena University Hospital, 07747 Jena, Germany; 9 Institute of Physical Chemistry and Abbe Center of Photonics, Helmholtzweg 4, 07743 Jena, Germany; 10 Department of Pharmacy, School of Medicine and Surgery, University of Naples Federico II, Via D. Montesano 49, 80131 Naples, Italy; 11 MRC Human Immunology Unit, Weatherall Institute of Molecular Medicine, University of Oxford, Headley Way, Oxford, OX39DS UK

**Keywords:** Anti-inflammatory therapy, 5-Lipoxygenase-activating protein, Poly(lactide-*co*-glycolide) (PLGA), Acetalated dextran, Polymer nanoparticles (NPs), Drug delivery

## Abstract

Leukotrienes are pro-inflammatory lipid mediators generated by 5-lipoxygenase aided by the 5-lipoxygenase-activating protein (FLAP). BRP-201, a novel benzimidazole-based FLAP antagonist, inhibits leukotriene biosynthesis in isolated leukocytes. However, like other FLAP antagonists, BRP-201 fails to effectively suppress leukotriene formation in blood, which limits its therapeutic value. Here, we describe the encapsulation of BRP-201 into poly(lactide-*co*-glycolide) (PLGA) and ethoxy acetalated dextran (Ace-DEX) nanoparticles (NPs), aiming to overcome these detrimental pharmacokinetic limitations and to enhance the bioactivity of BRP-201. NPs loaded with BRP-201 were produced via nanoprecipitation and the physicochemical properties of the NPs were analyzed in-depth using dynamic light scattering (size, dispersity, degradation), electrophoretic light scattering (effective charge), NP tracking analysis (size, dispersity), scanning electron microscopy (size and morphology), UV–VIS spectroscopy (drug loading), an analytical ultracentrifuge (drug release, degradation kinetics), and Raman spectroscopy (chemical attributes). Biological assays were performed to study cytotoxicity, cellular uptake, and efficiency of BRP-201-loaded NPs *versus* free BRP-201 to suppress leukotriene formation in primary human leukocytes and whole blood. Both PLGA- and Ace-DEX-based NPs were significantly more efficient to inhibit leukotriene formation in neutrophils *versus* free drug. Whole blood experiments revealed that encapsulation of BRP-201 into Ace-DEX NPs strongly increases its potency, especially upon pro-longed (≥ 5 h) incubations and upon lipopolysaccharide-challenge of blood. Finally, intravenous injection of BRP-201-loaded NPs significantly suppressed leukotriene levels in blood of mice in vivo. These results reveal the feasibility of our pharmacological approach using a novel FLAP antagonist encapsulated into Ace-DEX-based NPs with improved efficiency in blood to suppress leukotriene biosynthesis.

**Supplementary Information:**

The online version contains supplementary material available at 10.1007/s00018-021-04039-7.

## Introduction

5-Lipoxygenase-activating protein (FLAP) and microsomal prostaglandin E_2_ synthase-1 (mPGES-1) are considered as innovative drug targets [1] that accomplish the biosynthesis of the formation of pro-inflammatory lipid mediators from arachidonic acid (AA) involved in inflammation. Thus, FLAP assists 5-lipoxygenase (5-LO) in the generation of leukotrienes (LTs) that display potent chemotactic effects, activate pro-inflammatory leukocytes and constrict small vessels, while mPGES-1 catalyzes the transformation of cyclooxygenase-derived prostaglandin (PG)H_2_ to PGE_2_ that mediates pain and fever, and increases the vascular permeability [2, 3]. Our previous structure–activity relationship studies on benzimidazole-based dual inhibitors of FLAP and mPGES-1 revealed BRP-201 (5-{1-[(2-chlorophenyl)methyl]-2-{1-[4-(2-methylpropyl)phenyl]ethyl}-1H-benzimidazole-5-yl}-2,3-dihydro-1,3,4-oxadiazole-2-thione) as the most potent derivative [4]. However, BRP-201 and many other structurally different dual mPGES-1/FLAP inhibitors or FLAP antagonists suffer from loss of efficiency in biological/pharmacological relevant environments apparently due to strong unspecific protein binding based on their acidic and lipophilic structures [1, 5]. Moreover, these compounds are afflicted with low water solubility that may further hinder their bioactivity and bioavailability.

Employing bioavailability enhancement techniques, such as encapsulation of drugs into polymer-based nanoparticles (NPs), could improve the drug pharmacokinetics, thus increasing their bioactivity [6]. Numerous studies have documented the great potential of poly(lactide-*co*-glycolide) (PLGA) as drug delivery tool, however, only very few approaches have exploited the benefits of acetalated dextran as a promising alternative [7–9]. Dextran is an established biomaterial that has been used in medicine for various applications [10–12]. Besides its biocompatibility, the advantages of acetalated dextran are mainly its facile synthesis and the opportunity to tailor its degradation kinetics based on the degree of acetal functionalization. Thus, the main advantage of acetalated dextran is that it can be tailored with faster degradation rates compared to PLGA. Moreover, acetalated dextran can be processed into NPs in the same manner as polyesters to encapsulate hydrophobic drugs [7, 8]. Ethoxy acetalated dextran (Ace-DEX) is a dextran derivative with a safe toxicity profile, which degrades into dextran, ethanol, and acetone—all non-harmful metabolites. In addition, the Ace-DEX metabolites should not cause an acidic microenvironment, which is an undesirable effect often associated with the acidic metabolites of PLGA degradation [13]. Nevertheless, both polymers offer application opportunities, for example, the long-lasting PLGA is generally advantageous for developing sustained-release formulations (parenteral, subcutaneous, or intra-articular/muscular), whereas Ace-DEX can be developed into parenteral formulations with varying drug release profiles. Therefore, in this study, we document the formulation and characterization of Ace-DEX and PLGA NPs loaded with BRP-201, and further compare the efficiency of the NPs with that of the free BRP-201.

## Materials and methods

### Materials

Poly(*D,L*-lactide-*co*-glycolide) (PLGA) (Resomer RG502 H, M_w_ 7000 to 17.000 g mol^−1^, 50:50 co-polymer composition with carboxylic acid end groups), poly(vinyl alcohol) (PVA) (Mowiol 4–88, M_w_ 31.000 g mol^−1^), dimethyl sulfoxide (DMSO, anhydrous ≥ 99.9%), acetone, triethylamine (TEA), Rhodamine B isothiocyanate (mixed isomers), anhydrous pyridine (99.8%), and all other materials were purchased from Sigma Aldrich unless otherwise stated. Methanol and N,N-dimethyl acetamide (DMAc) were purchased from standard suppliers and were used without any further purification. THF was dried in a solvent purification system prior to use (SPS, Pure solv. EN, Innovative Technology). Lithium chloride (LiCl, 99%) was purchased from Acros Organics. Ethoxy acetalated dextran (Ace-DEX) was synthesized based on an established protocol where the hydroxyl groups of dextran were modified using 2-ethoxyprop-1-ene (95%, Fluorochem) (instead of 2-methoxypropene) [14]. Two batches of Ace-DEX were synthesized with the following properties: (#1) M_n_ 12,400 g mol^−1^, dispersity (Ð) of 1.68, and degree of substitution (DS) of acetal groups DS_cyclic_ = 1.81 and DS_acyclic_ = 0.49 and (#2) M_n_ 12,200 g mol^−1^, Ð = 1.73, DS_cyclic_ = 1.17, DS_acyclic_ = 0.96. The covalent coupling of Rhodamine B to Ace-DEX was achieved according to a previously published protocol [15]; M_n_ 22,400 g mol^−1^, Ð = 1.38, DS_cyclic_ = 1.82, DS_acyclic_ = 0.04, DS_Rho_ = 0.374 mg g^−1^. PLGA-Rhodamine B was synthesized according to the procedure described in Sect. 2.2; M_n_ = 10,900 g mol^−1^, Ð = 1.47, DS_Rho_ = 0.098 µg g^−1^. Pure water was used in all experiments for the NP preparation.

### PLGA-rhodamine B synthesis

In a Schlenk flask, PLGA (2 g, 0.29 mmol) and Rhodamine B isothiocyanate (27 mg, 0.05 mmol, 0.18 eq) were dissolved in anhydrous pyridine (20 mL) under Schlenk conditions. The dark red colored reaction solution was stirred at room temperature for 18 h, followed by stirring at 55 °C for 24 h. After cooling to room temperature, the reaction mixture was precipitated in a large excess of diethyl ether to get rid of the pyridine. The solid was collected by centrifugation and dissolved in THF. The resulting solution was precipitated in a large excess of methanol. The second precipitation step was repeated as often as necessary until the free dye was completely removed. The presence of free dye was checked between the precipitation steps by size exclusion chromatography measurements in DMAc and 0.21% LiCl with a UV–VIS detector at 555 nm. The obtained pellet from centrifugation was dried under vacuum, resulting in a pink colored powder (yield 58.2%).

### Nanoparticle formulation

Particles were formulated by nanoprecipitation using a syringe pump (Aladdin AL1000-220, World Precision Instruments) with a flow rate of 2 mL min^−1^. The organic solution was prepared by dissolving 25 mg of polymer (PLGA or Ace-DEX) in 5 mL acetone at 5 mg mL^−1^ polymer concentration. To load the NPs with the drug, 75 µL of 10 mg mL^−1^ BRP-201 solution (initially dissolved in DMSO) was added to the polymer solution and vortexed. The organic solution was infused into 36 mL of pure water (+ 100 µL of 0.01% TEA of pH = 10 for Ace-DEX NPs), while stirring at 800 rpm at room temperature. After the organic solution had been completely transferred into pure water, 4 mL of PVA 3% (w/v) solution was added to the NP dispersion. The NP dispersions were stirred at 800 rpm for 12–24 h for acetone to evaporate. To purify the NPs, the dispersions were centrifuged at 16.639×*g* for 60 min at 20 °C (Centrifuge 5804 R, Eppendorf). The NP pellets were then redispersed into 2.5 mL pure water (+ 100 µL of 0.01% TEA pH = 10 for Ace-DEX NPs). The NP dispersions were first vortexed for 10 s, sonicated in an ultrasound water-bath for 30 min, and stored overnight at 4 °C to allow for complete resuspension. The next day, NPs were lyophilized and the dried particles were stored at 4 °C. Rhodamine B-labeled Ace-DEX NPs were formulated according to the same protocol, except that the labelled Ace-DEX was mixed with pure Ace-DEX polymer in a 1:10 ratio. Ace-DEX NPs for in vivo application were prepared using a higher polymer and drug concentration, i.e., 15 mg mL^−1^ and 10% (w w^−1^) BRP-201 in relation to the polymer mass, respectively. The yield of the NPs was calculated according to the following formula:

$$Yield(\%)= \frac{(mass\, of \,NPs\, recovered-mass\, of\, found\, PVA)}{(mass\, of\, polymer+mass\, of \,drug)\, fed\, in\, the \,formulation}\times 100$$

### Dynamic light scattering (DLS) and electrophoretic light scattering (ELS)

DLS and ELS were used to estimate the hydrodynamic diameter (d_H_), polydispersity index (PDI), and zeta-potential (ζ-potential) of the NPs (Zetasizer Nano ZS, Malvern Instruments). The laser wavelength of the Zetasizer was *λ* = 633 nm and all measurements were performed at 25 °C and a 173° backscattering angle. The d_H_ and PDI of NPs were measured after evaporation of acetone, after centrifugation of NPs, and after lyophilization, while the ζ-potential was measured only after lyophilization of the NPs. The concentration of the measured NPs after purification and after lyophilization was 4 mg mL^−1^ for Ace-DEX-based NPs and 7 mg mL^−1^ for PLGA-based NPs. The DLS procedure for d_H_ and PDI estimations consisted of five measurements each consisting of 5 runs of 30 s, with an equilibration time of 30 s before and between measurements. The ELS procedure for the ζ-potential consisted of three measurements with 3 runs with 30 s equilibration time before and between measurements.

### Nanoparticle tracking analysis (NTA)

A NanoSight NS500 (Malvern Panalytical) was used to determine the NP sizes in terms of HD. The lyophilized NPs were redispersed in pure water and measured at the following concentrations:

50 µg mL^−1^ for PLGA-based NPs, 10 µg mL^−1^ for Ace-DEX-based NPs, 1 µg mL^−1^ for BRP-201 drug precipitates. For each NP sample, five videos of 60 s acquisition time were captured at room temperature with instrument settings adjusted as reported in Table S1 (SI).

### Scanning electron microscopy (SEM)

A Sigma VP field emission scanning electron microscope (Carl-Zeiss AG) was used to obtain the particle images. The microscope was operated with the InLens detector at a 6 kV acceleration voltage. 5 µL of NP dispersions were pipetted on mica substrates and air-dried. Before the measurement, samples were coated with a thin layer of platinum (4 nm) via sputter coating (CCU-010 HV, Safematic). ImageJ was used to estimate the NP size from 300 and 500 particles per image, for Ace-DEX and PLGA NPs, respectively.

### UV–VIS spectroscopy

Encapsulation efficiency (EE) and loading capacity (LC) of the BRP-201-loaded particles were determined using a UV–VIS plate reader (Infinite M200 Pro Platereader, Tecan Group Ltd.). The samples were prepared as follows: three aliquots of 200 µL NP dispersion were lyophilized; the NP powder was accurately weighed and then redissolved in 200 µL of UV-grade DMSO. The solutions were pipetted on a Hellma Quartz 96-well plate and measured at *λ* = 316 nm, with 3 × 3 multiple reads per well and a 2000 µm well border. A calibration curve of BRP-201 was obtained in a concentration range of 0.48–250 µg mL^−1^, and the following formulas were used to calculate LC and EE, respectively.

$$LC= \frac{mass\, of\, drug\, recovered}{mass\, of\, particle\, recovered} \times 100$$$$EE=\frac{LC }{LC\, theoretical } \times 100$$

UV–VIS spectroscopy was also used to determine the content of the surfactant (%, w w^−1^) in the lyophilized NPs according to a previously published protocol [16]. The concentration of the redispersed NPs was 3 mg mL^−1^ for the unlabeled NPs and approx. 0.5 mg mL^−1^ for the labeled NPs.

### Degradation of NPs

Particle degradation was measured by DLS according to a previously described protocol [15]. The normalized value of the measured count rate (in percentage) was used to plot the apparent degradation profile of the NPs against time.

### Raman spectroscopy

Samples were placed on CaF_2_ slides (Crystal GmbH, Germany) for Raman characterization, either as solid samples or drop coated (2 × 5 µL and allowed to dry at ambient conditions). Raman spectra were recorded on an upright Raman microscope (α300, Witec) with a 600 l/mm grating. The Raman excitation laser (488 nm, Witec) was focused with a Nikon 100 × NA 0.8 objective onto the sample resulting in 1 mW in the sample plane. Under these conditions, the best spatial resolution was around 300 nm. Single Raman spectra were recorded from bulk polymeric samples with an integration time of 1 s per spectrum. Raman maps were recorded with a step size of 125 nm utilizing the same conditions. Statistical analysis was performed using GNU R. Spectral pre-processing involved spike removal, baseline correction (polynomial baseline fitting) and normalization (area normalization). False color Raman images were generated by plotting the intensity ratio of the Raman bands at 1620 cm^−1^ (C=N vibration in BRP-201) and 1450 cm^−1^ (C-H deformation band for Ace-DEX NPs) and to the C=O stretching band at 1760 cm^−1^ (for PLGA NPs), respectively.

### Drug release from the NPs

NPs were incubated in 0.05 mM acetate (pH = 4.5) or 0.05 mM phosphate buffer (pH = 7.4) at 37 °C for the following times: Ace-DEX NPs for 0.5, 1, 2, 5, 20, and 144 h; PLGA NPs for 0, 1, 7, 20, and 30 days. Additionally, control samples consisting of NPs in pure water were studied with the same experimental settings but without incubating the samples at 37 °C. All NP formulations were investigated at a concentration of 0.5 mg mL^−1^.

Sedimentation velocity experiments were conducted using an Optima Analytical Ultracentrifuge (AUC) (Beckmann Coulter Instruments, Brea, CA) with an An-50 Ti eight-hole rotor. Rotor position eight was used as the counterbalance, enabling the optical module calibration. All ultracentrifuge cells contained double sector Epon centerpieces with a 12 mm solution optical path length and sapphire windows. The corresponding sectors were filled with approx. 440 µL pure solvent as a reference and approx. 420 µL of sample solution. Scans were acquired in four-minute intervals by using the interference optics and absorbance optical detection in terms of optical density (OD) at a wavelength of 316 nm, *i.e.* being representative of the encapsulated drug BRP-201. In total, 480 scans with a four-minute time interval (32 h) were recorded at a rotor speed of 1500 rpm. After that, the rotor speed was subsequently accelerated to 42,000 rpm for the investigation of potentially present smaller species in the supernatant for a further 24 h (or 18 h for Ace-DEX NPs with 0.5–20 h incubation in acetate buffer and the respective control sample), also providing 480 scans (or 360 scans) with a 3 min time interval. All measurements were performed at 22 °C. Every fourth scan was considered for data evaluation. The recorded sedimentation velocity data were numerically analyzed with SEDFIT and the ls–g*(s) model considering non-diffusing species [17].

### Cell isolation and cell culture

Leukocyte concentrates were prepared from peripheral blood obtained from healthy adult male and female donors that received no anti-inflammatory treatment for the last 10 days (Institute of Transfusion Medicine, Jena University Hospital). The approval for the protocol was given by the ethical committee of the Jena University Hospital and all methods were performed in accordance with the relevant guidelines and regulations. For isolation of neutrophils and monocytes, the leukocyte concentrates were mixed with dextran (from *leuconostoc spp.* M_W_ ~ 40,000, Sigma Aldrich) for sedimentation of erythrocytes and the supernatant was centrifuged on lymphocyte separation medium (Histopaque^®^-1077, Sigma Aldrich). Contaminating erythrocytes in the pelleted neutrophils were removed by hypotonic lysis (using water). Neutrophils were then washed twice in ice-cold phosphate-buffered saline (PBS) pH 7.4 and finally resuspended in PBS pH 7.4. The peripheral blood mononuclear cell (PBMC) fraction on top of the lymphocyte separation medium was washed with ice-cold PBS pH 7.4 and seeded in cell culture flasks (Greiner Bio-one) for 1.5 h (37 °C, 5% CO_2_) in PBS pH 7.4 with Ca^2+^/Mg^2+^ to isolate monocytes by adherence. For differentiation and polarization of monocytes to M1 macrophages, we followed published procedures [18]. Thus, adherent monocytes were treated with 20 ng mL^−1^ granulocyte macrophage-colony stimulating factor (Peprotech) for 6 days in RPMI 1640 supplemented with 10% fetal calf serum (FCS), 2 mmol L^−1^ l-glutamine, penicillin (100 U mL^−1^) and streptomycin (100 µg mL^−1^), and further incubated with 100 ng mL^−1^ LPS and 20 ng mL^−1^ interferon-γ (Peprotech) for 48 h to obtain M1 macrophages. Correct polarization and purity of macrophages was routinely checked by flow cytometry (BD LSR Fortessa, BD Biosciences, Heidelberg, Germany) as reported [19] using the following antibodies: FITC anti-human CD14 (2 µg/test, clone M5E2, BD Biosciences), PE anti-human CD54 (1 µg/test, clone HA58, BD Biosciences), APC-H7 anti-human CD80 (0.25 µg/test, clone L307.4, BD Biosciences), PE-Cy7 anti-human CD163 (2 µg/test, clone RM3/1, Biolegend, San Diego, CA, USA), PerCP-eFluor710 anti-human CD206 (0.06 µg/test, clone 19.2, BD Biosciences).

### Fluorescence dye-labeled nanoparticle uptake in neutrophils

Time-dependent uptake of fluorescence dye-labeled NPs by neutrophils was analyzed by flow cytometry and confocal fluorescence microscopy. Adherent neutrophils (2 × 10^6^) were incubated for 30 min or 3 h with LPS (1 µg mL^−1^) or vehicle, and then with 0.5 mg mL^−1^ labeled NPs (PLGA-Rho[BRP-201] or Ace-DEX-Rho[BRP-201]) for indicated time points. Cells were washed once with PBS pH 7.4 containing 0.5% BSA and incubated with PBA-E (PBS pH 7.4 with 0.5% BSA, 2 mM EDTA and 0.1% sodium azide). Neutrophils containing fluorescently stained NPs were analyzed by flow cytometry using BD LSR Fortessa (BD Biosciences). The violet laser (405 nm) in combination with 610|20 filters for Rhodamine B-labeled NPs were used for flow cytometric analysis. Data were analyzed using FlowJo X Software (BD Biosciences). For confocal imaging, 25 mm glass coverslips were first sonicated in double-distilled water for 20 min, and subsequently dried with pressured air and plasma-cleaned for 30 s. Neutrophils were diluted to 650.000 cells mL^−1^ in PBS, and NP solution in PBS was added 3 min before imaging to a final concentration of 25 µg mL^−1^. Images were taken on a Zeiss LSM 980 confocal microscope with ZEN 3.0 blue software suite at 37 °C and on a Zeiss LSM 880 microscope at 37 °C and 5% CO_2_. On both setups, a Plan-Apochromat 63x/1.40 Oil-objective was used. For image analysis, ImageJ / Fiji were used [20, 21]. Figures were composed using FigureJ [22]. Time-line imaging was specifically realized by taking transmission brightfield images identifying the cell borders using the transmission T-PMT detector, and confocal fluorescence images of the NPs were recorded with a 561 nm laser source and detected between 570 and 680 nm with a GaAsP-PMT (pinhole size 1 airy unit). One three-dimensional image stack was taken every 5 min.

### Evaluation of 5-lipoxygenase product formation in human neutrophils

For evaluation of the effects of test items on 5-LO product formation in human neutrophils, cells (5 × 10^6^ mL^−1^) were pre-incubated with BRP-201 NPs (Ace-DEX[BRP-201], PLGA[BRP-201]) and non-loaded NPs (Ace-DEX, PLGA) for different periods at 37 °C. Cells were then stimulated with 2.5 µM Ca^2+^-ionophore A23187 (Cayman) for 10 min, and the incubation was stopped with 1 mL ice-cold methanol containing 200 ng mL^−1^ PGB_1_ as internal standard. Samples were subjected to solid phase extraction and formed 5-LO products (LTB_4_, trans-isomers of LTB_4_, 5-hydroperoxyeicosatetraenoic acid (5-HETE)) were separated and analyzed by RP-HPLC as previously described [23].

### Determination of lipid mediator signature profiles in human monocyte-derived macrophages

Human monocyte-derived M1 macrophages (M1-MDM; 2 × 10^6^ cells) were seeded in 6-well-plates and pre-incubated for 15 min, 5 h or 20 h with BRP-201 or NPs (Ace-DEX, PLGA, Ace-DEX[BRP-201], PLGA[BRP-201]) at 37 °C. The cells were subsequently incubated with *Staphylococcus aureus*-conditioned medium (SACM, *S. aureus* strain “6850”, 24 h culture, OD = 0.05) for 180 min. The reaction was stopped with ice-cold methanol containing deuterium-labeled internal standards (d8-5*S*-HETE, d4-LTB_4_, d5-LXA_4_, d5-RvD2, and d4-PGE_2_; 500 pg each). Samples were kept at – 20 °C for one day to allow protein precipitation. After centrifugation (2000×*g*, 4 °C, 10 min), 8 mL acidified water was added (final pH = 3.5) and samples were subjected to solid phase extraction using RP-18 columns and the lipid mediators (LMs) were analyzed by ultra-performance liquid chromatography-tandem mass spectrometry (UPLC-MS–MS) using an Acquity UPLC system (Waters) and a QTrap 5500 Mass Spectrometer (Sciex) equipped with an electrospray ionization source exactly as described before [18].

### Determination of lipid mediator profiles in human whole blood

Freshly withdrawn whole blood in Li-heparin Monovettes (Sarstedt) from healthy adult donors that had not received any anti-inflammatory treatment the last 10 days was provided by the Institute of Transfusion Medicine, Jena University Hospital. The blood was incubated for different periods with either BRP-201 or NPs (Ace-DEX, Ace-DEX[BRP-201], PLGA, PLGA[BRP-201]) and stimulated with pathogenic *E. coli* (O6:K2:H1; 1 × 10^9^ cells per mL blood) for 180 min. The reaction was stopped with ice-cold methanol containing the deuterium-labeled internal standards d8-5S-HETE, d4-LTB_4_, d5-LXA_4_, d5-RvD2, and d4-PGE_2_ (500 pg, each). Samples were kept at − 20 °C for 1 day to allow protein precipitation. After centrifugation (2000×*g*, 4 °C, 10 min) 8 mL acidified water was added (final pH = 3.5). The samples were subjected to solid phase extraction and analyzed by UPLC–MS–MS as described previously [18], see above (Sect. Determination of lipid mediator signature profiles in human monocyte-derived macrophages).

### Inhibition of LTB_4_ formation in murine blood in vivo

Adult (6–8 weeks) male CD1 mice (Charles River, Calco, Italy) were housed at the animal care facility of the Department of Pharmacy of the University of Naples “Federico II” and kept under controlled environment (i.e., temperature 21 ± 2 °C and humidity 60 ± 10%) and provided with normal chow ad water ad libitum. Mice were allowed to acclimate for 4 days prior to experiments and were subjected to 12 h light/dark schedule. Treatments were conducted during the light phase. The experimental procedures were approved by the Italian Ministry and carried out in accordance with the EU Directive 2010/63/EU and the Italian DL 26/2014 for animal experiments and in compliance with the ARRIVE guidelines and Basel declaration including the 3R concept. Mice (*n* = 6/group) received an injection of 200 µL consisting of 7 mg mL^−1^ (46 mg kg^−1^) Ace-DEX[BRP-201] NPs containing 4.6 mg kg^−1^ BRP-201 or the respective amount of Ace-DEX NPs in saline intravenously (i.v.) into the tail vein. After 3 h, zymosan (1 mg per mouse in 0.5 mL saline) was injected intraperitoneally (i.p.) to induce inflammation [24]. After another 4 h, mice were euthanized in a saturated CO_2_ atmosphere, and blood (0.7–0.9 mL) was collected by intracardiac puncture through insertion of a 1 mL syringe with a needle of 22 gauge (Carl Roth GmbH & Co. KG, Karlsruhe, Germany) using citrate as anticoagulant (3.8%, w v^−1^), immediately after euthanization. Plasma was obtained by centrifugation of the blood at 800×*g* at 4 °C for 10 min and immediately frozen for further analysis of LTB_4_ via UPLC–MS–MS as described above.

### Statistics

Results are expressed as mean ± standard error of the mean (S.E.M) of n observations, where n represents the number of experiments with cells from separate donors, performed on different days. The sample size was chosen empirically based on previous studies to ensure statistical power [15, 18, 19]. Datasets were analyzed by GraphPad Prism 9.2.0 (GraphPad, La Jolla, CA, USA). One-way ANOVA and Tukey’s multiple comparisons test were used for statistical analysis in case of one different independent variable influences one continuous dependent variable. Multiple t-test was used for comparison of different concentrations of two groups. Two-way ANOVA was used for statistical analysis in case of two different categorical independent variables influence one continuous dependent variable, as indicated. The criterion for statistical significance is *P < 0.05; **P < 0.01; ***P < 0.001.

## Results and discussion

### Nanoparticle formulation and characterization

For the encapsulation of BRP-201, we used two polymer materials with different degradation kinetics, i.e. PLGA and Ace-DEX that can be processed into NPs via nanoprecipitation applying the same formulation parameters (solvent, drug load, concentration, water-organic ratio, surfactant concentration) [15]. Previously, we used methoxy acetalated dextran (Ac-DEX) for the encapsulation of BRP-187, another mPGES-1/FLAP inhibitor and demonstrated the advantage of the encapsulating material [15]. However, upon degradation, Ac-DEX decomposes into dextran, acetone and methanol [25]. To omit the formation of methanol—although demonstrated to be non-toxic at concentrations usually applied for drug delivery—an ethoxy acetal derivative of dextran was used for encapsulation in this study. The ethoxy acetal derivative releases the less toxic ethanol instead of methanol upon degradation (SI, Fig. S1). This can be an advantage in case of higher doses of NPs are administered or in case of prolonged treatment times required during chronic treatments [14].

**Fig. 1:**
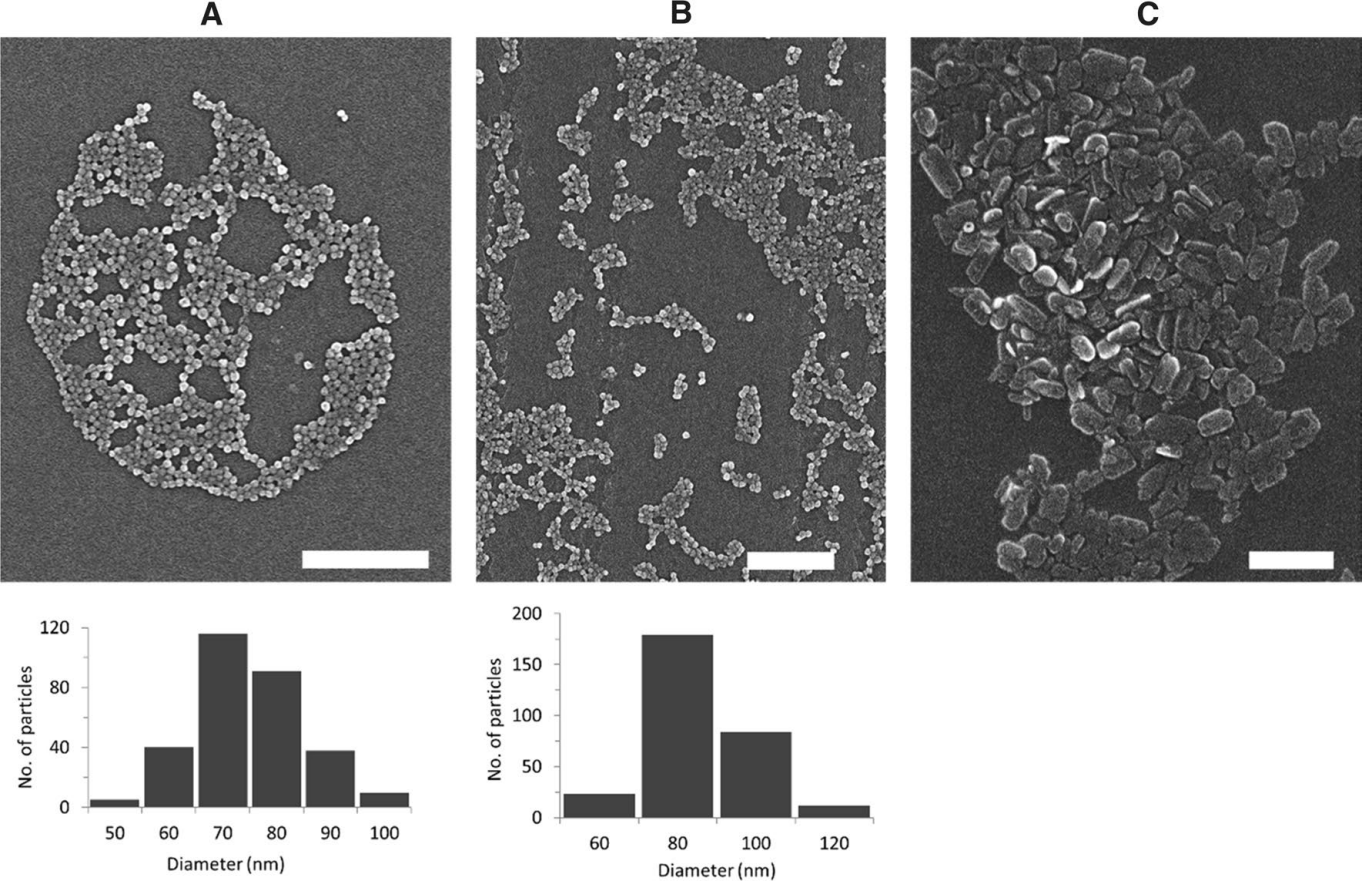
Scanning electron micrographs of the NPs: Ace-DEX (**A**), Ace-DEX[BRP-201] (**B**), and BRP-201 precipitates (**C**). Histograms were generated from ImageJ measurements (*n* = 300). The scale bars are 1 µm

NPs with 3% (w w^−1^) BRP-201 were first analyzed by DLS, which revealed that particles feature a size of around 150 to 200 nm with narrow size distribution (PDI < 0.2) (with the exception of Ace-DEX-Rho). All NPs had a negative ζ-potential of – 20 to – 30 mV, indicating stable NP formulations (Table 1). Similar insights were obtained from NTA measurements, where PLGA[BRP-201] showed a mixture of differently sized species in the formulation and a higher concentration of species larger than 200 nm (SI, Fig. S3B). A control experiment of precipitating the free drug without polymer into water showed that BRP-201 formed non-spherical particle-like precipitates of around 250 to 300 nm in hydrodynamic size. The size of the NPs as well as the formation of the drug precipitates were also confirmed by SEM (Fig. 1, and Fig. S2 in SI). The drug-formed precipitates were slightly larger in size and PDI than the polymeric NPs (SI Table S2, Fig. 1, Fig. S2 in SI). According to the SEM, the PLGA[BRP-201] formulation showed a mixture of uniformly distributed spherical NPs and particle-like structures of the drug (SI, Fig. S2B and C). Similar results were obtained from NTA measurements, where PLGA[BRP-201] showed a mixture of differently sized species in the formulation and a higher concentration of species larger than 200 nm (SI Fig. S3B, Table S2). NPs prepared with 3% (w w^−1^) BRP-201 revealed optimal properties regarding size distribution and encapsulation efficiency (EE). However, to reach satisfactory doses in vivo, the preparation protocol for Ace-DEX NPs was optimized in order to further increase the drug loading. Both polymer and drug concentration fed in the formulation were increased to 15 mg mL^−1^ and 10% (w w^−1^), respectively. The resulting NPs remained within the desired size range of 150–270 nm, with a similar yield but with an EE of 100% (SI Table S3). Although the drug loading was considerably higher (92 µg mg^−1^ NPs) when 10% (w w^−1^) BRP-201 was fed in the formulation, drug precipitates were clearly present (Fig. 1C). Nevertheless, investigations of the particles via MADLS revealed that when NP dispersions were mixed with 0.9% NaCl, only 20% of the particle population was around 3 µm (SI Table S4, Fig. S13). In this case, Ace-DEX-based NPs were within a size range that pose a low risk of irritation at the injection site, [26] and are below the suggested limits of 5 μm for the upper particle size for injectable dispersions (SI Table S4) [27, 28]. Also, drug-loaded NPs measured after 28 days (stored at 4 °C) revealed that the particle size remained below the suggested limit of 5 μm (SI Table S5).

**Table 1:** Summary of the physicochemical properties of NPs loaded with 3% (w/w) BRP-201

Formulations with 3% BRP-201	Purified NP suspension		Lyophilized NPs			Yield	PVA	EE	LC
	d_H_ (nm)	PDI	d_H_ (nm)	PDI	ζ (mV)	(%)	(%, w/w)	(%)	(%, w/w)
PLGA	158 ± 5	0.11 ± 0.02	166 ± 12	0.07 ± 0.02	− 24 ± 4	67	2 ± 0	−	−
PLGA[BRP-201] 3%	174 ± 5	0.15 ± 0.02	182 ± 7	0.13 ± 0.02	− 22 ± 3	71	1 ± 0	75 ± 11	2.2 ± 0.3
PLGA-Rho	177 ± 11	0.13 ± 0.00	168 ± 11	0.09 ± 0.01	− 25 ± 1	59	18 ± 1	−	−
PLGA-Rho[BRP-201] 3%	201 ± 6	0.14 ± 0.01	191 ± 7	0.12 ± 0.03	− 23 ± 2	51	15 ± 1	79 ± 5	2.3 ± 0.2
Ace-DEX	140 ± 20	0.10 ± 0.05	172 ± 17	0.23 ± 0.08	− 19 ± 6	41	3 ± 1	−	−
Ace-DEX[BRP-201] 3%	154 ± 27	0.08 ± 0.02	128 ± 32	0.09 ± 0.03	− 24 ± 6	39	1 ± 0	69 ± 16	2.0 ± 0.5
Ace-DEX-Rho	185 ± 21	0.10 ± 0.02	391 ± 49	0.43 ± 0.05	− 16 ± 1	58	10 ± 1	−	−
Ace-DEX-Rho[BRP-201] 3%	214 ± 11	0.08 ± 0.01	182 ± 7	0.08 ± 0.01	− 27 ± 1	56	6 ± 0	70 ± 1	2.1 ± 0.1
BRP-201 precipitates	336 ± 79	0.23 ± 0.15	317 ± 44	0.18 ± 0.09	− 23 ± 2	−	−	−	−

d_H_ (Z-average), PDI and zeta-potential (ζ) measured by DLS and ELS, where *n* represents number of batches (PLGA and Ace-DEX *n* = 8; Rho-labeled NPs *n* = 3; BRP-201 precipitates *n* = 5); EE and LC determined by UV–VIS spectroscopy (*n* = 9)

In general, the formulation parameters were efficient since the EE for all NPs was > 70% (Table 1). Based on the spectroscopic quantification, the amount of BRP-201 in 1 mL of NPs dissolved in DMSO was around 318 µM for PLGA[BRP-201] and 139 µM for Ace-DEX[BRP-201] for NPs prepared with 3% (w w^−1^) drug. Meanwhile, NPs prepared with 10% (w w^−1^) drug contained on average 1.3 mM BRP-201 in 1 mL of NPs dissolved in DMSO. Considering that the IC_50_ of BRP-201 for inhibition of FLAP and mPGES-1 are 0.04 and 0.42 µM, respectively, stable NP dispersions with drug concentration of several hundred- to thousand-folds higher compared to the drugs’ IC_50_ values were achieved. This is important for the biodistribution of the NPs in vivo since in general less than 15% of the injected dose of NPs reaches the intended target [29–31]. In addition, administering high doses of NPs reduces liver clearance and prolongs the circulation of NPs, since high doses of NPs overwhelm the receptors of Kupffer cells in the liver [32].

The amount of residual PVA was found to be on average 10% (w w^−1^) in all NP dispersions, with the exception of the dye-labeled PLGA NPs, which retained 15 to 18% (w w^−1^) of residual surfactant (Table 1). A higher amount of residual PVA was observed for NPs with 10% (w w^−1^) BRP-201 (SI Table S3), since here the initial polymer concentration fed in the formulation was threefold higher when compared to the NPs with 3% (w w^−1^) BRP-201 (Table 1). Note that surfactants are necessary to preserve the stability of the NPs, however, excess amounts should be removed from the dispersion to diminish their influence on the NP cellular uptake processes [33].

### Degradation of nanoparticles

The NP degradation behavior was studied in acetate (pH 4.5) and phosphate buffer (pH 7.4), both at 37 °C. The count rate in DLS corresponds to the size and number of particles scattering the laser beam, i.e. a high count rate indicates numerous NPs scattering that light [34]. A decreasing count rate over time at fixed scattering detector conditions indicates the degradation of the NPs due to a decreasing size and/or their number [15].

**Fig. 2:**
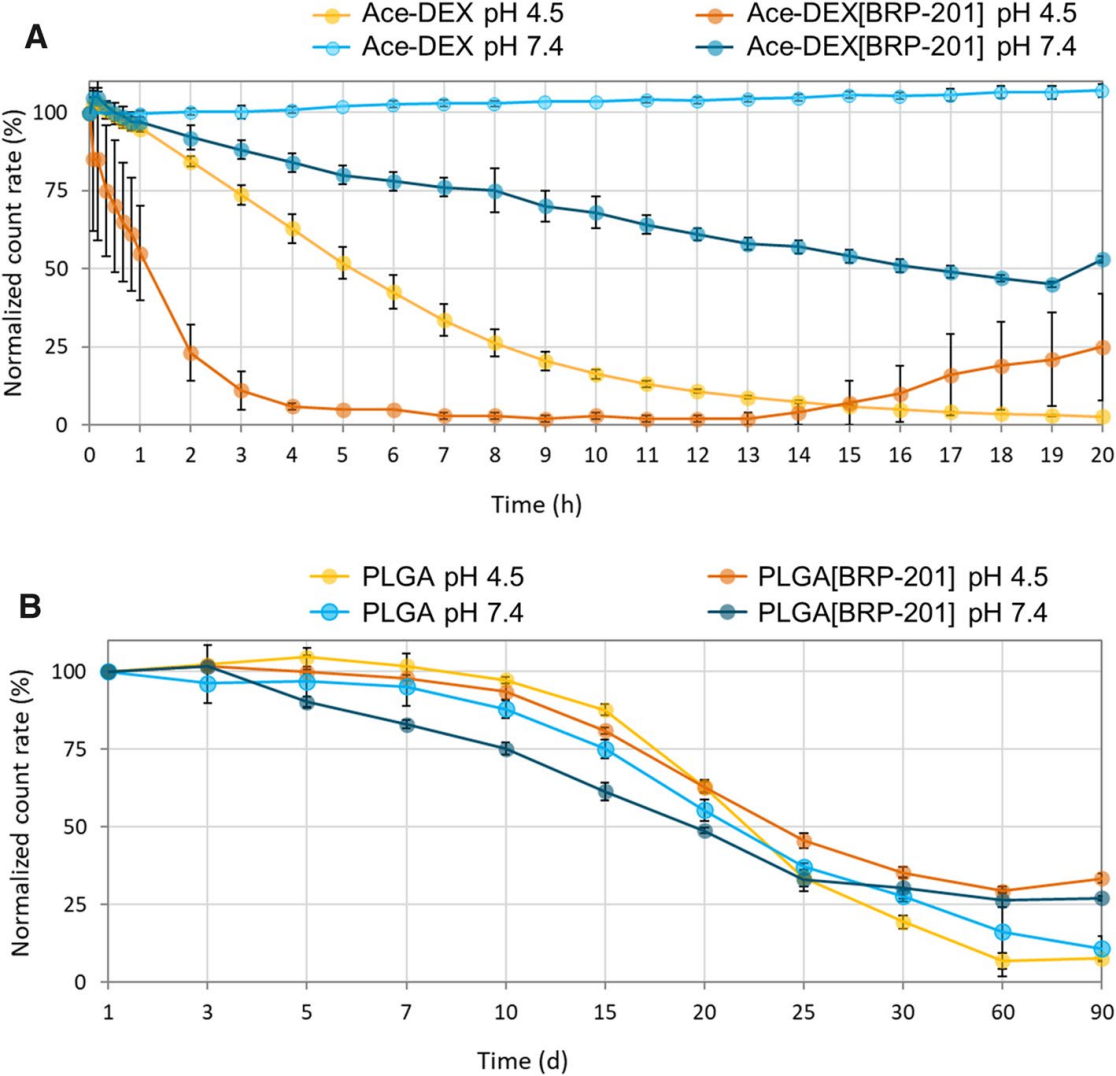
Apparent decrease in count rates of PLGA and Ace-DEX NPs at 37 °C incubated in 0.05 mM acetate buffer (pH 4.5) and 0.05 mM phosphate buffer (pH 7.4), as measured by DLS at fixed scattering detector settings (*n* = 3). The derived count rate on DLS was measured at pre-determined times, and plotted as normalized value against the derived count rate at timepoint 0 of incubation with buffer solution

Figure 2A shows that for Ace-DEX[BRP-201] NPs the count-rate decreased by about 50% after 50 min when incubated at pH 4.5, whereas at pH 7.4, the count-rate decreased by 50% of the NPs after approx. 20 h. Furthermore, the drug-loaded Ace-DEX NPs apparently degraded faster than their unloaded counterparts, regardless of the medium pH value, suggesting that BRP-201 may accelerate erosion of the NPs. The results further imply that at low pH, Ace-DEX NPs presumably show higher apparent erosion, which is observed first with an immediate increase in the size and PDI of the NPs (SI, Fig. S4, A and B). This could indicate aggregation of the NPs, occurring due to the fast hydrolysis of the acyclic acetals at their surfaces, followed by a further degradation towards the interior of NPs proceeding mainly from the slower hydrolysis of the cyclic acetals. The relatively fast degradation initiated by the cleavage of acetal groups is similar to polyketal-based polymers [14, 35]. In addition, the apparent erosion of the Ace-DEX NPs is faster under acidic conditions than at neutral pH value, indicated by a progressive decrease in the size of NPs (SI, Fig. S4, A), where after 20 h the size of the NPs decreased by about 40 nm for Ace-DEX[BRP-201].

On the other side, PLGA NPs followed a sigmoidal progression of count-rate decreases, typical for polyester-based polymers (Fig. 2B) [36, 37]. A 50% decrease in the count rate of the PLGA[BRP-201] NPs was observed between day 20 and 25 of incubations at both pH 4.5 and 7.4 (Fig. 2B). However, it should be noted that endogenous enzymes (esterases) or other solution components in vitro and in vivo could accelerate the degradation of PLGA, and hence its degradation rate could be faster than that observed in a buffer-only medium [38]. NPs labeled with Rhodamine B showed similar apparent degradation profiles as the non-labeled particles (SI, Figs. S5 and S6).

### Drug release from the NPs

**Fig. 3:**
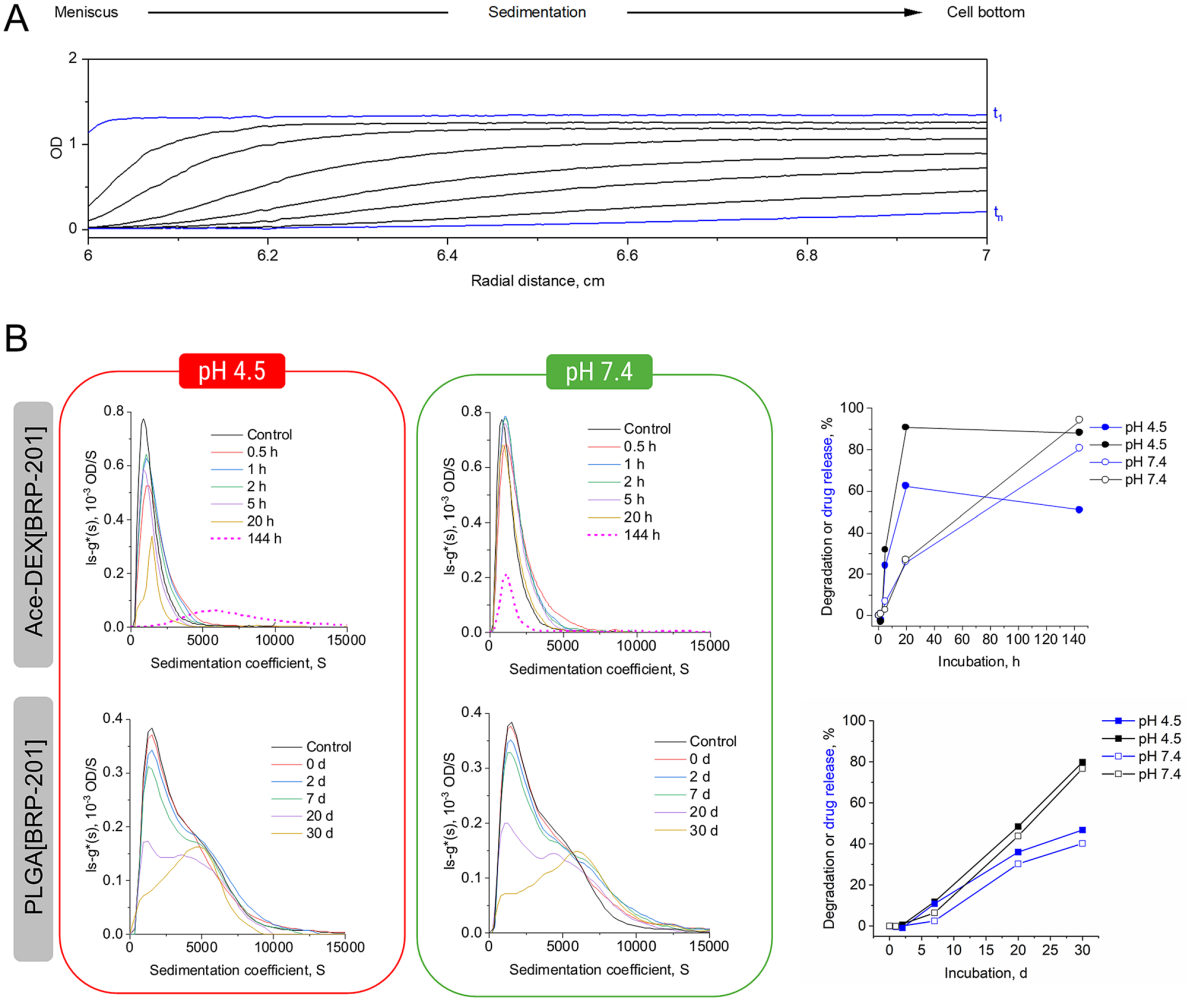
**A** Selected sedimentation velocity profiles observed via absorbance detection at *λ* = 316 nm (in terms of OD) of Ace-DEX[BRP-201] NPs incubated for 0.5 h at pH 4.5 and 37 °C with an early scan, t_1_, and a late scan, t_n_, highlighted in blue. **B** Differential distributions of sedimentation coefficients, ls—g*(s), from sedimentation analysis of sedimentation velocity experiments at a rotor speed of 1500 rpm, observed via absorbance detection at *λ* = 316 nm (in terms of OD) of the NPs after incubation in 0.05 mM acetate buffer (pH 4.5) (left), and 0.05 mM phosphate buffer (pH 7.4) (middle) at 37 °C; the control refers to drug-loaded NPs in water stored at 4 °C prior to the measurement. (Right) Apparent NP degradation and/or drug release was determined from the RI and UV signal intensities (in terms of integrated areas under the differential distribution curves), respectively, at pH 4.5 and pH 7.4. The values were calculated by normalization of areas under the curve from each time at measurement against the recorded areas under the curves from the NPs incubated for 0.5 h only

Ace-DEX[BRP-201] and PLGA[BRP-201] NPs were investigated via sedimentation velocity experiments with an AUC, using multi-detection for the observation of the apparent NP erosion processes and drug behavior (via RI detection and absorbance detection) [39, 40]. Ace-DEX[BRP-201] NPs were studied on a timescale between 0.5 and 144 h (Fig. 3, Figs. S9 and S10 in the SI). The sedimentation velocity experiments enable analytical tracking of sedimenting material according to its size in the centrifugal field. Thereby, material successively moves from the meniscus to the cell bottom at increased timescales captured with every scan by the detection modules (Fig. 3A). From these time- and radially-resolved sedimentation profiles, the differential distributions of sedimentation coefficients can be obtained, representative of the population of sedimenting material and its amount. The differential distribution of sedimentation coefficients, ls-g*(s), of the Ace-DEX[BRP-201] NPs in water (control) displayed the highest signal intensity in both detection mode (Fig. 3B and Fig. S9 (top)). The samples investigated at pH 4.5 and pH 7.4 showed differential distributions of sedimentation coefficients with lower intensities at increased timescales. Ace-DEX[BRP-201] NPs incubated for 144 h at pH 4.5 showed a distinct distribution of higher sedimentation coefficients, which indicates the presence of a distinct population of species (Fig. 3B, left), apparently absent at pH 7.4 (Fig. 3B, right). However, this distinct population at higher sedimentation coefficients was not seen that significant with the RI detection (SI, Fig. S9). Thus, the larger population of species that were only observed with the absorbance detection hint toward the formation of nanoprecipitates of poorly soluble BRP-201 after the complete NP erosion at pH 4.5.

Based on areas calculated from the differential distributions of sedimentation coefficients obtained from the absorbance detection in terms of OD, a time-dependent drug release from Ace-DEX[BRP-201] NPs at both pH values was clearly evident (Fig. 3B, right). Ace-DEX[BRP-201] NPs incubated at pH 4.5 released the encapsulated drug faster than at pH 7.4. After 5 h, the NPs released more than 20% of drug at pH 4.5, and less than 10% at pH 7.4. After 20 h, the NPs released about 60% of the drug at pH 4.5, whereas at pH 7.4 only about 25% of the drug was released (Fig. 3B, right).

Investigations of PLGA[BRP-201] NPs revealed similar features of the differential distributions of sedimentation coefficients, ls-g*(s), from the absorbance detection (in terms of OD) and RI detection for samples mixed with buffer of pH 4.5 and pH 7.4 (solutions were primarily heated to 37 °C) immediately before the measurement (Fig. 3B left and middle (bottom); SI Fig. S9 (bottom)). The differential distributions of sedimentation coefficients based on absorbance detection clearly exposed a shoulder toward higher sedimentation coefficients becoming more evident and ultimately dominating the distribution after 30 days of incubation at both pH values (Fig. 3B, left and middle (bottom)). This shoulder indicates an abundance of larger species unveiled by active separation of the species in the AUC. In comparison to the results from the absorbance detection, the RI detection revealed a similar decrease of the signal intensity with increased incubation times regardless of the pH values, with a barely visible shoulder toward larger sedimentation coefficients, even after 30 days of incubation (SI, Fig. S19, left and middle (bottom)). This could be explained by the less sensitive RI detection when compared to the absorbance detection when considering the drug nanoprecipitates. The same BRP-201 nanoprecipitates were also observed in the SEM and indicated by the NTA as well (vide supra). Apparently, the drug tends to form nanosized objects in solution that are not encapsulated within the polymeric matrix (particularly forming during erosion). Those species have a distribution of sedimentation coefficients exceeding those of the NPs as seen by the other analytical techniques as well. The degradation, as well as the drug release appeared slightly faster for NPs incubated at pH 4.5, as was similarly observed for Ace-DEX[BRP-201] NPs (Fig. 3B, right). However, here the degradation and drug release started after approx. 7 days of incubation and reached approx. 50% apparent degradation and 30% drug release after 20 days of incubation at both pH values.

The derived apparent degradation data obtained from the AUC where also in accordance with the count-rate DLS data (representative of apparent degradation) at the same chosen timescales for both types of NPs (SI, Fig. S10). Nicely, the vastly different timescales (hours and days) for a decrease of count rates is mirrored with the sedimentation velocity AUC data (Fig. 3B, right). Further investigations performed at higher centrifugal speeds revealed the presence of smaller species in the supernatant that corresponded to the PVA surfactant (used in the formulation process), also showing a response faster for acetate buffer at pH 4.5 than for phosphate buffer at pH 7.4 for the Ace-DEX[BRP-201] NPs (SI, Fig. S11 (top)) while appearing less pronounced for the PLGA[BRP-201] NPs (SI, Fig. S11 (bottom)). After 144 h of incubation, the signal intensity of PVA increased by approx. threefold for Ace-DEX[BRP-201] at both pH conditions, while simultaneously, the RI intensities of the NPs decreased by approx. tenfold at pH 4.5 and 18-fold at pH 7.4 (SI, Fig. S12, A). Similar trends were observed for PLGA-based NPs (SI, Fig. S12, B), and also for other medical NPs [40].

### Chemical elucidation of nanoparticles via Raman spectroscopy

**Fig. 4:**
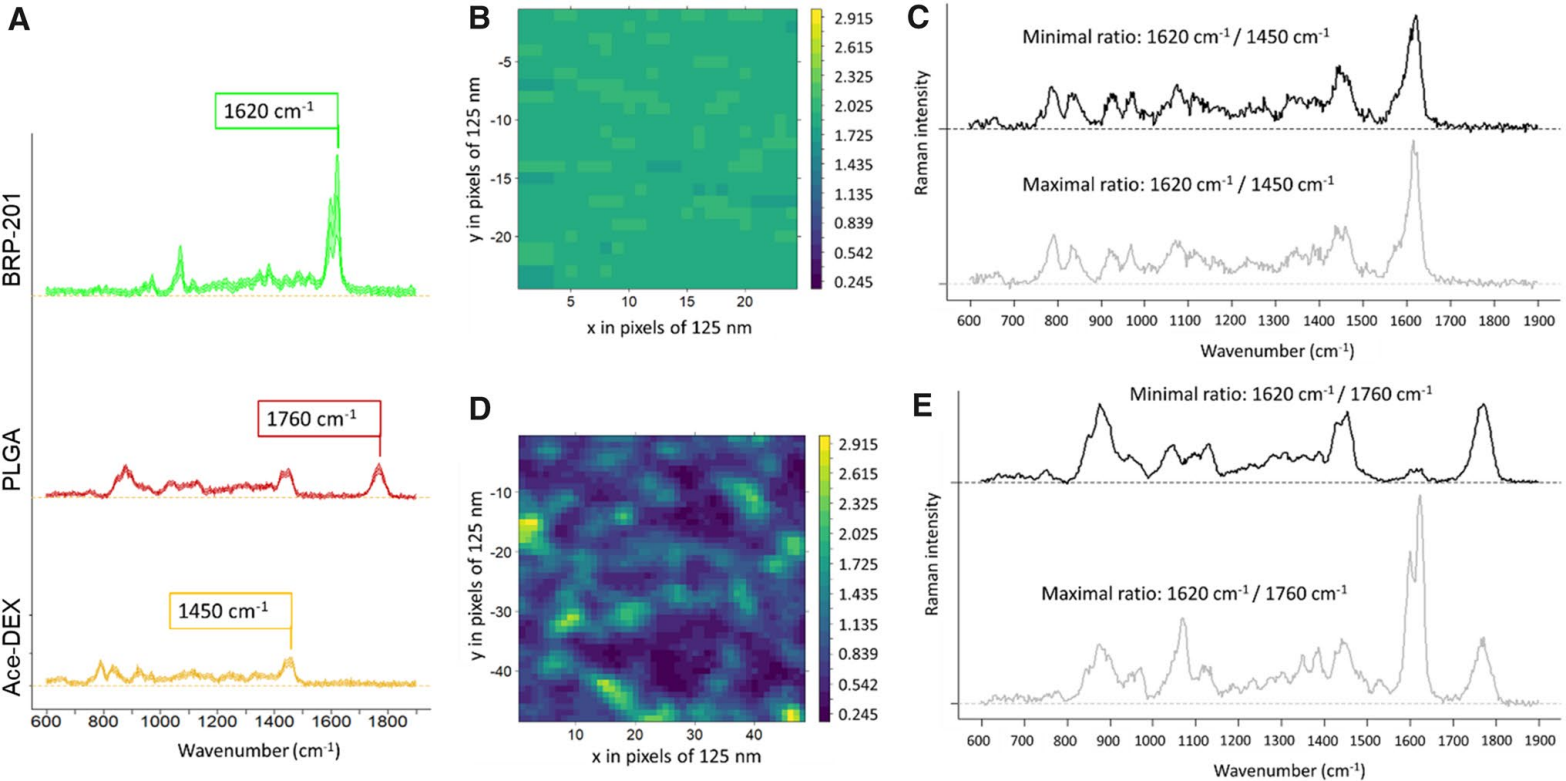
**A** Mean Raman spectra with standard deviation of pure nanomaterial: BRP-201 precipitates (green), PLGA NPs (red) and Ace-DEX NPs (yellow); spectra were shifted on the y-axis for clarity; **B**, **D** false color Raman images calculated from the ratio of the Raman intensities at 1620 cm^−1^ (BRP-201) and **B** 1450 cm^−1^ (Ace-DEX) or **D** 1760 cm^−1^ (PLGA), respectively; the color codes the intensity ratio; axis (x,y) are labeled in pixels that have dimensions of 125 × 125 nm^2^ each; **C**, **E** depict individual Raman spectra representing the extreme ratios from the false color Raman maps in B and D, respectively

Raman spectroscopy was employed to investigate the chemical properties of individual formulations, and in particular, to investigate if free drug is present as precipitates in the Ace-DEX[BRP-201] and PLGA[BRP-201] formulations. The Raman mean spectra of the two polymers and the drug (Fig. 4) revealed characteristic structural features of the substances. For BRP-201, the most prominent Raman band was at 1620 cm^−1^, which can be assigned to the C=N vibration in the benzimidazole ring, and was not overlapping with Ace-DEX (C-H deformation band around 1450 cm^−1^) and PLGA (C=O stretching vibration at 1760 cm^−1^) bands. Thus, it was used to visualize the relative abundance of the drug in the NPs. The false color Raman image of the PLGA[BRP-201] formulation (Fig. 4D) showed a non-homogeneous distribution of PLGA and BRP-201, where distinct regions with high abundance of BRP-201 were observed (green regions in Fig. 4D, and light grey Raman spectrum in 4E). The size of the regions in the false color Raman image was in the range of the spatial resolution limit, which means that indicated features must be 300 nm or smaller. Other regions were rich in PLGA and showed only a low BRP-201 content (blue regions in Fig. 4D, and dark grey Raman spectrum in 4E). For Ace-DEX[BRP-201] formulations, no such inhomogeneities were observed (Fig. 4B). Here, the drug and polymer were relatively equally distributed as noted from the small range in the false color intensity scale (Fig. 4B), and from the Raman spectra (Fig. 4C).

Furthermore, suspensions of Ace-DEX[BRP-201] NPs were incubated for 48 h at either pH 4.5 or pH 7.4, and subsequently samples were prepared by drop coating and analyzed. Only few larger accumulations of solid particles were found in the dried samples (bright field images, SI Fig. S7). Raman spectra of the dried NP samples (SI, Fig. S8) showed almost exclusively spectral contributions from BRP-201 at different concentrations. Spectral signals from Ace-DEX were not visible in the analyzed sample region. These results confirm that after 48 h, Ace-DEX[BRP-201] NPs were completely degraded, leaving only BRP-201 as detectable material in the Raman spectra.

### NP uptake in neutrophils

**Fig. 5:**
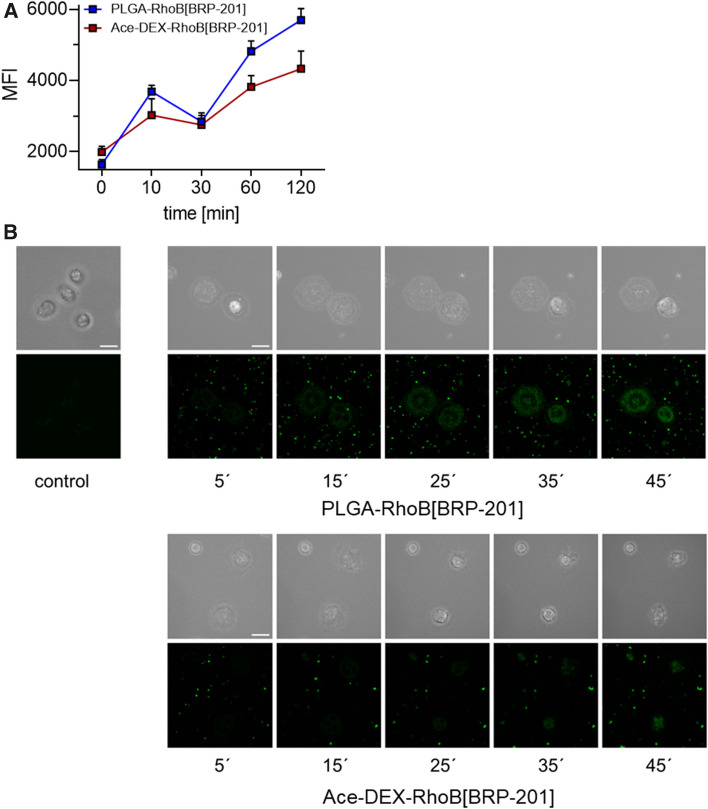
Uptake of Rhodamine B-labeled NPs in human neutrophils. Neutrophils (2 × 10^6^) were pre-incubated with 1 µg mL^−1^ LPS for 30 min or vehicle and then incubated with 0.5 mg mL^−1^ (PLGA-RhoB[BRP-201] or Ace-DEX-RhoB[BRP-201]) NPs for the indicated time points at 37 °C. (A) Mean fluorescence intensity (MFI) of NPs in the cell, measured by flow cytometry. (B) Neutrophils (3 × 10^5^) were seeded on coated coverslips. Images were taken on a Zeiss LSM 880 and a Zeiss LSM 980 microscope at 37 °C and 5% CO_2_. A nanoparticle-solution was added to a final solution of 25 µg mL^−1^ before imaging. The top row represents the z-projected brightfield images of neutrophils, bottom row the single slide confcoal fluorescence images of Rhodamine B emission of NPs, highlighting cellular NP uptake. Scale bars = 10 µm.

Neutrophils are a major source for inflammatory lipid mediator (LM) production in the blood stream and were evaluated for NP uptake. We monitored the uptake of Rhodamine B-labeled NPs over 120 min for MFI measurement (Fig. 5A) and 45 min for imaging using transmission brightfield and confocal fluorescence microscopy (Fig. 5B) for identifying cells and NPs, respectively. Both types of NPs were sufficiently taken up by neutrophils with PLGA NPs being superior to Ace-DEX NPs (Fig. 5). However, the lower MFI of Ace-DEX NPs could also be a result of the faster decomposition inside the cell since the polymer is rapidly degraded at lower pH values. This hypothesis fits to the identical uptake until 60 min, where Ace-DEX NPs start to decompose (Fig. 2A).

### Evaluation of free and encapsulated BRP-201 on cell viability in human primary leukocytes

**Fig. 6:**
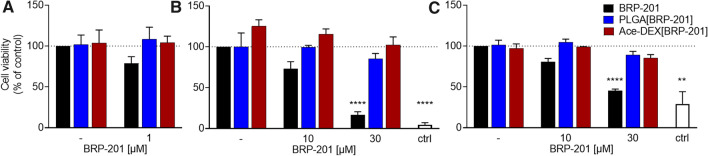
Cytotoxicity analysis. (**A**) Neutrophils resuspended in PBS pH 7.4 containing 0.1% glucose were incubated with vehicle (“–“), BRP-201, empty NPs (“–“) or NPs loaded with BRP-201 for 5 h at 37 °C. Then, cell viability was assessed by trypan blue staining with a ViCell XR device (Beckman Coulter). **B**, **C** M1-MDM in RPMI 1640 medium were incubated with vehicle, control (3 µM staurosporine), BRP-201, empty NPs or NPs loaded with BRP-201 for 24 h at 37 °C and cell viability was assessed using MTT assay (**B**) or LDH release assay (**C**). Values are given as percentage of control (DMSO), data are means ± S.E.M., n = 3. For Statistics a one-way ANOVA and Tukey’s multiple comparisons test were performed

We next analyzed free BRP-201, empty NP, and NPs loaded with BRP-201 for potential cytotoxic effects in human neutrophils and M1-MDM. After 5 h incubation of neutrophils, BRP-201 (1 µM) slightly but not significantly reduced cell viability (trypan blue staining) to 85%, while no detrimental effects on cell viability were detectable for the NPs (Fig. 6A). Although neutrophils are a suitable cell model to study inhibition of 5-LO product formation, their short half-life hampers the suitability for long term (> 6 h) studies. Therefore, we also used M1-MDM that also generate substantial amounts of 5-LO products, representing relevant target cells with a pro-inflammatory phenotype, with the advantage of being suitable for prolonged incubations (up to 48 h). Using two cell viability assays that address mitochondrial functionality (MTT assay, Fig. 6B) and membrane integrity (LDH-assay, Fig. 6C) we found that in M1-MDM incubated for longer periods (24 h), cytotoxic effects of BRP-201 at 30 µM were obvious, a concentration that is 60- to 80-fold higher than the effective concentration to inhibit LM formation in these cells [4]. When BRP-201 was encapsulated in NPs, this detrimental effect was abolished, implying that the encapsulation of BRP-201 in Ace-DEX and PLGA NPs increases the compatibility of BRP-201.

### Inhibition of 5-LO product formation by free and encapsulated BRP-201 in human primary leukocytes

**Fig. 7:**
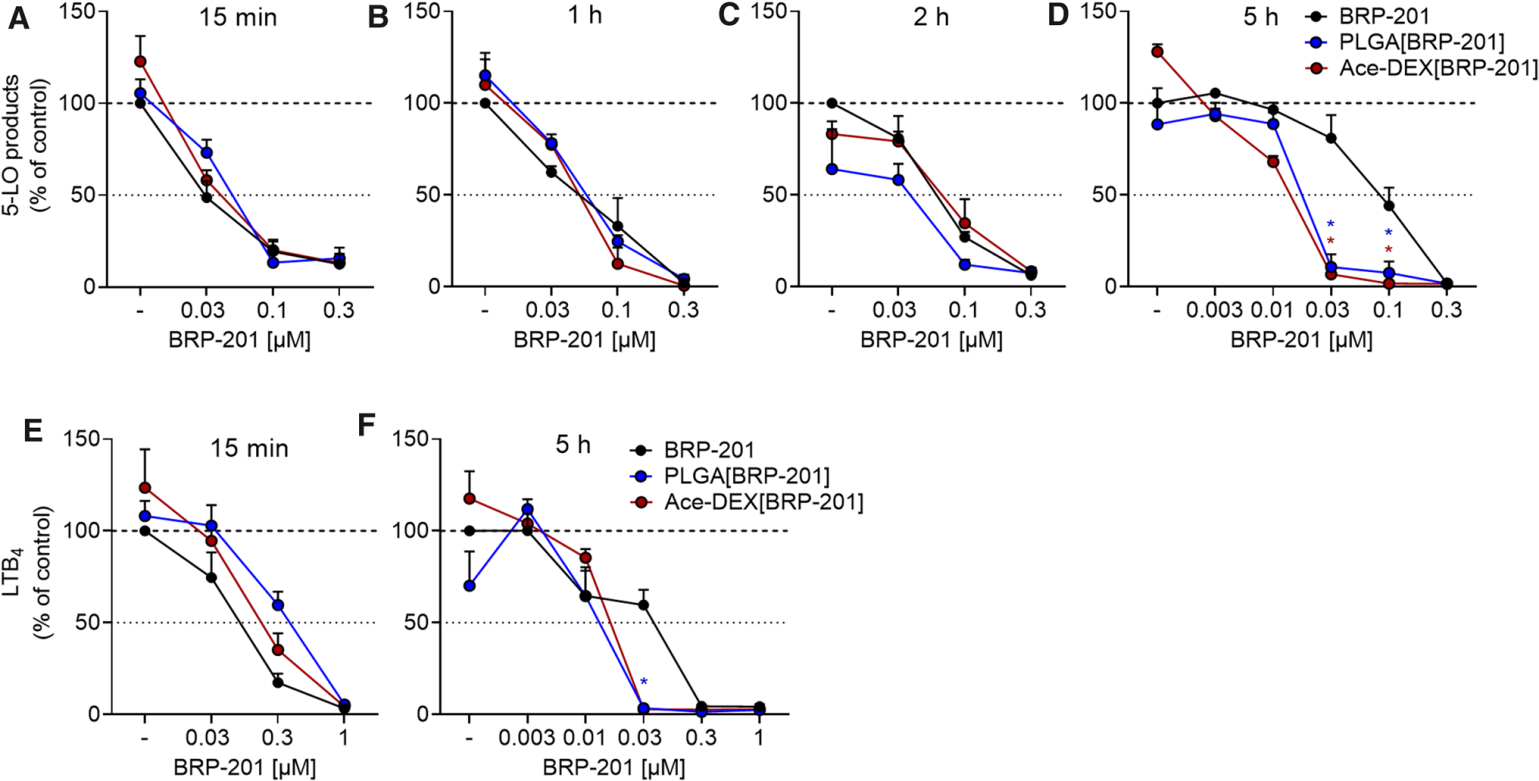
Inhibition of 5-LO product formation by free and encapsulated BRP-201 in isolated leukocytes. **A**–**D** Neutrophils were pre-incubated with vehicle, BRP-201 or BRP-201-loaded NP made from PLGA or Ace-DEX for 15 min (**A**), 1 h (**B**), 2 h (**C**) or 5 h (**D**) at 37 °C and then stimulated with 2.5 µM A23187. After 10 min, the reaction was stopped, and 5-LO products were extracted via solid phase extraction (SPE) and analyzed with HPLC. Values are given as 5-LO products (LTB_4_, trans-LTB_4_, and 5-HETE) in percentage of control (vehicle, DMSO = 100%). **E**, **F** M1-MDM were pre-incubated with vehicle, BRP-201 or BRP-201-loaded NP made from PLGA or Ace-DEX for 15 min **E** or 5 h **F** at 37 °C and then stimulated with 0.1% *S. aureus*-conditioned medium (SACM). After 3 h at 37 °C, the reaction was stopped and LTB_4_ was analyzed by UPLC–MS–MS. Values are given as percentage of the vehicle control. Data are means ± S.E.M., *n* = 3. For statistical analysis, two-way ANOVA and multiple *t* tests were performed

To evaluate the efficiency of BRP-201 as free drug and encapsulated into NPs for inhibition of cellular 5-LO product formation, we first used human neutrophils as primary innate immune cells with high capacities to generate 5-LO products involving FLAP [41]. Freshly isolated neutrophils were pre-incubated with the test items for various periods (i.e., 15 min, 1 h, 2 h, and 5 h) and then stimulated for FLAP-dependent 5-LO product formation using 2.5 µM A23187 for 10 min. When BRP-201 as well as drug-loaded NPs (PLGA and Ace-DEX) were pre-incubated for short periods (i.e., 15 min up to 2 h), the potency of free and of encapsulated BRP-201 was comparable with > 80% inhibition of 5-LO product formation at 0.1 µM and IC_50_ values of approx. 30 nM (Fig. 7A–C). However, after 5 h preincubation, encapsulated BRP-201 in NPs was more efficient versus the free drug. Thus, at 30 nM, corresponding to the IC_50_ of free BRP-201 under standard assay conditions in neutrophils [4], 5-LO product formation is potently reduced by Ace-DEX[BRP-201] or PLGA[BRP-201] down to 6 ± 4% and 11 ± 7% remaining activity, while free BRP-201 was much less potent and 81 ± 21% 5-LO product formation still remained (Fig. 7D); the IC_50_ values for free BRP-201, Ace-DEX[BRP-201] and PLGA[BRP-201] after 5 h preincubation were 86, 13, and 18 nM. These data indicate that BRP-201 loses potency during prolonged (> 2 h) preincubation of neutrophils. However, potent inhibition of 5-LO product formation by BRP-201 is achieved and maintained in neutrophils over time due to encapsulation of the drug in Ace-DEX- and PLGA-based NPs.

Next, we performed experiments with pro-inflammatory M1-MDM stimulated with 0.1% *S. aureus*-conditioned medium (SACM) to induce 5-LO product formation within 3 h, according to [42]. In analogy to A23187-activated neutrophils, the results with M1-MDM confirmed the beneficial effect of BRP-201 encapsulation in NPs versus free drug on 5-LO product formation after long-term incubation. Thus, after 5 h of preincubation, the amount of LTB_4_ was reduced by 30 nM encapsulated BRP-201 down to 4 ± 0.8% for Ace-DEX[BRP-201] and 3 ± 0.7% for PLGA[BRP-201] NPs while free BRP-201 impaired LTB_4_ formation only to 59 ± 8.3% (Fig. 7F). In contrast, preincubation of M1-MDM with Ace-DEX[BRP-201] or PLGA[BRP-201] NPs for only 15 min was slightly less efficient to inhibit 5-LO product formation versus the free drug (Fig. 7E), possibly due to retarded supply of BRP-201 from NPs into the cells.

### Inhibition of 5-LO product formation by BRP-201 and NPs in human whole blood

**Fig. 8:**
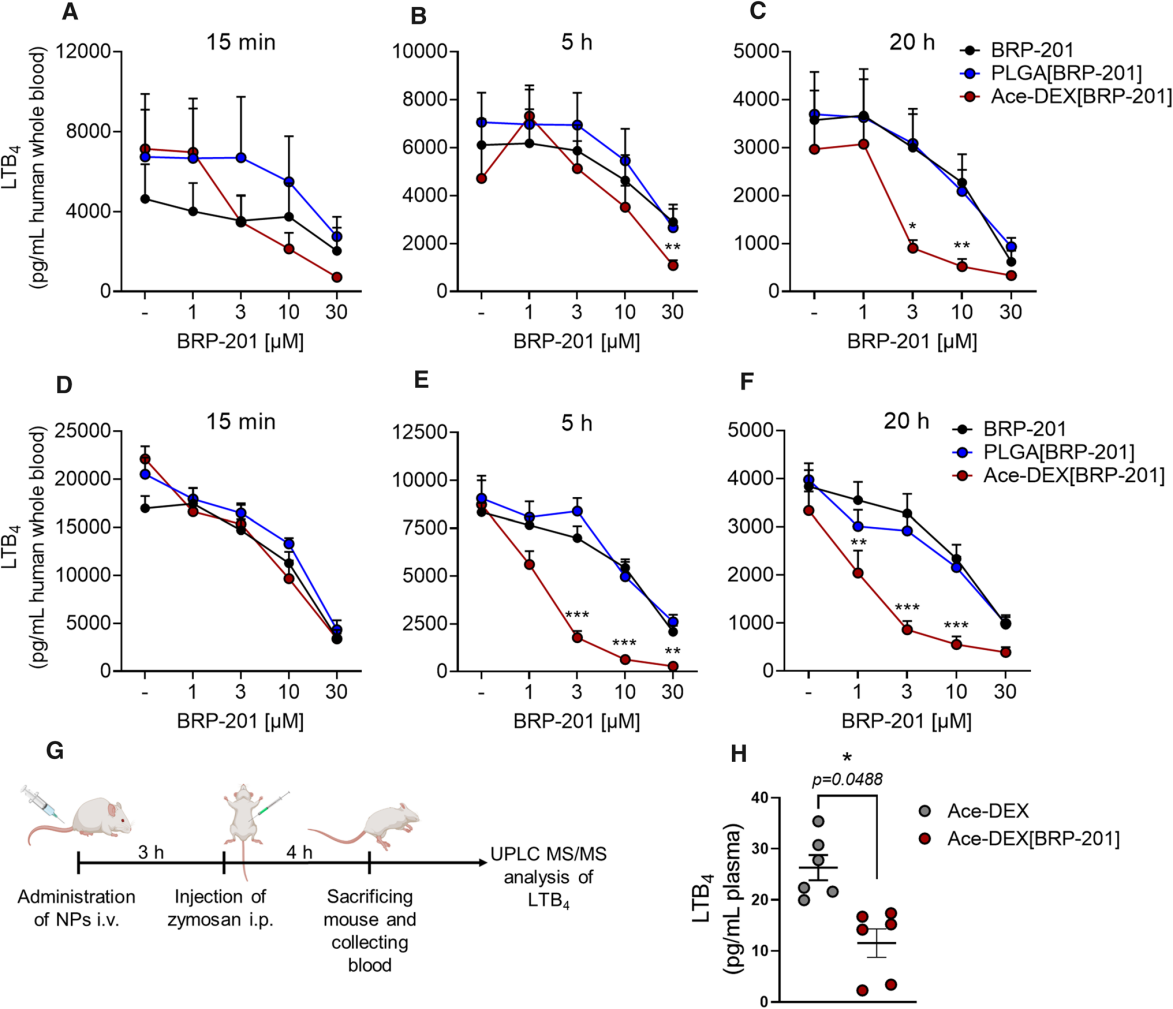
Efficient inhibition of LTB_4_ production by encapsulated BRP-201 in human whole blood and in blood of mice in vivo. **A**–**C** Freshly withdrawn blood was pre-incubated with vehicle, BRP-201, empty NPs or NPs loaded with BRP-201 for **A** 15 min, **B** 5 h or **C** 20 h prior to stimulation with *E. coli* (O6:K2:H1; 10^9^ bacteria per 1 mL blood) for 3 h at 37 °C. **D**–**F** Freshly withdrawn blood was first pre-treated with 100 ng mL-1 LPS for 24 h and then pre-incubated with vehicle, BRP-201, empty NPs or NPs loaded with BRP-201 for **D** 15 min, **E** 5 h or **F** 20 h prior to stimulation with E. coli (O6:K2:H1; 10^9^ bacteria per 1 mL blood) for 3 h at 37 °C. The incubations were terminated by addition of 2 mL ice-cold methanol and samples were analyzed for LTB_4_ by UPLC–MS–MS as described. Data are means ± S.E.M., *n* = 6. **G** Timeline of mouse experiments. **H** Mice (*n* = 6) received empty NPs or NPs loaded with BRP-201 by i.v. injection into the tail vein, 3 h prior zymosan administration (i.p.). After another 4 h, blood was collected by intracardiac puncture and plasma was prepared. LTB_4_ was extracted from plasma by SPE and analyzed via UPLC MS–MS. For statistics, a two-way ANOVA, multiple t-tests and a ratio paired t-test were performed, *n* = 6

One critical issue of FLAP as target for anti-inflammatory therapy is the need of rather lipophilic antagonists that compete with AA for binding to FLAP [43–45]. Due to these structural requirements most FLAP inhibitors display strong unspecific protein and membrane binding with overall poor bioavailability in more complex experimental settings such as whole blood, where excess of plasma protein as well as non-targeted cells (e.g. platelets, erythrocytes) are present and impair the efficiency of the compound. Thus, we tested the potency of free and encapsulated BRP-201 under varying preincubation periods (15 min, 5 h and 20 h) in *E. coli*-exposed human whole blood, where neutrophils and monocytes are the major sources for LT formation. Compared to the high potency of BRP-201 in isolated neutrophils resuspended in PBS pH 7.4 (IC_50_ = 0.03 ± 0.013 µM), the compound significantly lost efficiency in whole blood with IC_50_ = 14.7 ± 3.8 µM at 15 min and 10.4 ± 2.1 µM at 20 h pre-incubation (Fig. 8). However, when encapsulated into Ace-DEX, the potency of BRP-201 was improved about fivefold with an IC_50_ = 2.8 ± 0.5 µM, in particular after prolonged (20 h) preincubation periods (Fig. 8C), while pretreatment for 15 min (Fig. 8A) or 5 h (Fig. 8B) was less effective (IC_50_ = 4.2 ± 0.9 and 8.2 ± 2.1 µM). In contrast, encapsulation of BRP-201 into PLGA did not significantly enhance its potency (Fig. 8A–C).

Next, we aimed at creating a pro-inflammatory environment within the whole blood incubations by pre-treatment of the blood with LPS for 24 h prior to addition of the free drug or drug-loaded NPs for various pre-incubation periods (15 min, 5 h and 20 h) and subsequent stimulation by addition of *E. coli* for another 3 h. Under these experimental conditions the LT-producing neutrophils and monocytes become activated by LPS similarly like at inflammatory sites. Again, free BRP-201 moderately suppressed LT formation, comparable as in the absence of LPS and independent of the preincubation period, with IC_50_ values in the range of 10–18 µM (Fig. 8D). But when the LPS-treated blood was pre-preincubated with Ace-DEX[BRP-201] NPs for 5 or 20 h, the efficiency of BRP-201 to inhibit LT formation strongly improved by about a factor of 10 (IC_50_ = 1.6 ± 0.3 and 1.9 ± 0.4 µM, respectively), and was even superior as compared to blood devoid of LPS exposure (Fig. 8E and F). For example, while 20 h preincubation with 1 µM reduced LTB_4_ formation down to 86 ± 9% in the absence of LPS, in LPS-treated blood the LTB_4_ production was lowered down to 51 ± 9% (Fig. 8F).

Finally, we injected Ace-DEX[BRP-201] NPs i.v. via the tail vein of mice to test if encapsulated BRP-201 could suppress LTB_4_ production in blood in vivo*.* Mice received a suspension of 46 mg kg^−1^ Ace-DEX[BRP-201] NPs, which corresponds to a dose of 4.6 mg kg^−1^ BRP-201, or 46 mg kg^−1^ empty Ace-DEX NPs without BRP-201. After 3 h, zymosan was injected (i.p.) in order to induce an inflammatory condition with increased LTB_4_ formation in the blood, which was analyzed after another 4 h by UPLC-MS–MS (Fig. 8G). As can be seen from Fig. 8H, Ace-DEX[BRP-201] NPs significantly impaired the levels of LTB_4_ as compared to empty Ace-DEX NPs devoid of drug, supporting the in vivo delivery performance of the formulation following i.v. injection.

## Conclusion

This study was designed to improve the bioactivity of BRP-201, a potent FLAP antagonist that, however, loses potency in human blood when compared to isolated leukocytes—a common feature of almost all FLAP antagonists [43–45]. Such loss of efficiency of BRP-201 and other FLAP inhibitors in blood have been attributed to unspecific but strong plasma protein or cell membrane binding with consequently low efficacy in in vivo experiments and subsequent clinical trials [44, 45]. To tackle this problem, we used PLGA and Ace-DEX as biocompatible polymers to encapsulate BRP-201 into biodegradable NPs to mitigate plasma protein binding, and thus, to improve drug delivery to FLAP in the target cells within the blood. We optimized the NPs and comprehensively characterized their physicochemical properties, thus supporting the beneficial features of the final formulations. In general, we found that the encapsulation of BRP-201 was more promising in Ace-DEX NPs than PLGA NPs, which was due to the formation of drug precipitates in PLGA NPs as indicated by SEM, AUC, and Raman mapping. Thus, Ace-DEX NPs present a more homogenous formulation than PLGA NPs with the desirable feature of achieving a fast release of BRP-201. The design of the NP carriers has also implications for inflammatory cell models, i.e., BRP-201 loaded in Ace-DEX but not in PLGA was able to potently suppress LT formation in human whole blood, although both Ace-DEX[BRP-201] NPs and PLGA[BRP-201] NPs caused more potent inhibition of 5-LO product formation in isolated neutrophils and M1-MDM after prolonged pre-incubation *versus* the free drug, without any significant differences between the two polymers. Of interest, in addition to enhancing the LT-inhibitory potency, encapsulation of BRP-201 lowers cytotoxic effects after prolonged incubations with MDM. Under conditions where monocytes and neutrophils are activated by LPS to mimic an inflammatory environment, such as at sites of inflammation, the potency of Ace-DEX[BRP-201] NPs in blood is even further improved. In fact, when given *i.v.* to mice that were challenged with zymosan to induce an inflammatory reaction, Ace-DEX[BRP-201] NPs significantly lowered the LTB_4_ levels in blood in vivo, supporting the feasibility of our approach and its benefit in anti-inflammatory therapy. In summary, we designed polymer-based nanoformulations of BRP-201, which have been characterized in detail for their physicochemical properties, drug loading and release, cytotoxicity, cellular uptake and desired bioactivity, eventually overcoming the detrimental challenges in the development of FLAP antagonists. Further optimization for scaling-up the batches beyond the laboratory bench may provide sufficient material for more advanced studies related to drug testing in preclinical and clinical settings.

### Supplementary Information

Below is the link to the electronic supplementary material.

Supplementary file1 (DOCX 11135 KB)

## Data Availability

The datasets used and/or analyzed during the current study are available from the corresponding authors on reasonable request.

## Abbreviations

5-LO: 5-Lipoxygenase
5S-HETE: 5-Hydroxyeicosatetraenoic acid
AA: Arachidonic acid
Ace-DEX: Ethoxy acetalated dextran
AUC: Analytical ultracentrifuge
BSA: Bovine serum albumin
d_H_: Hydrodynamic diameter
DLS: Dynamic light scattering
DMAc: Dimethyl acetamide
DMSO: Dimethyl sulfoxide
ELS: Electrophoretic light scattering
EDTA: Ethylenediaminetetraacetic acid
EE: Encapsulation efficiency
FCS: Fetal calf serum
FLAP: 5-Lipoxygenase-activating protein
IC50: Half maximal inhibitory concentration
LC: Loading capacity
LDH: Lactate dehydrogenase
LM: Lipid mediator
LPS: Lipopolysaccharide
LT: Leukotriene
MADLS: Multi-angle dynamic light scattering
MDM: Monocyte-derived macrophages
MFI: Mean fluorescence intensity
mPGES-1: Microsomal prostaglandin E2 synthase-1
MTT: 3-(4,5-Dimethylthiazol-2-yl)-2,5-diphenyltetrazolium bromide
NPs: Nanoparticles
NTA: Nanoparticle tracking analysis
PBA-E: PBS with 0.5% BSA, 2 mM EDTA and 0.1% sodium azide
PBMC: Peripheral blood mononuclear cell
PBS: Phosphate-buffered saline
PDI: Polydispersity index
PG: Prostaglandin
PLGA: Poly(lactide-*co*-glycolide)
PVA: Poly (vinyl alcohol)
RP-HPLC: Reversed phase-high performance liquid chromatography
RvD2: Resolvin D2
SACM: *S. aureus*-conditioned medium
SEM: Scanning electron microscope
SPE: Solid phase extraction
TEA: Triethylamine
THF: Tetrahydrofuran
UPLC-MS/MS: Ultra performance liquid chromatography tandem mass spectrometry
