# Supplementary material for: Ethoxy acetalated dextran-based nanocarriers accomplish efficient inhibition of leukotriene formation by a novel FLAP antagonist in human leukocytes and blood
Source: Cell Mol Life Sci. 2021 Dec 31;79(1):40. doi: 10.1007/s00018-021-04039-7 (PMC8966466; doi:10.1007/s00018-021-04039-7)
Supplement: Supplementary file 1 — Supplementary file1 (DOCX 11135 KB) [file 18_2021_4039_MOESM1_ESM.docx]

**Ethoxy acetalated dextran**-based nanocarriers accomplish efficient inhibition of leukotriene formation by a novel FLAP antagonist in human leukocytes and blood

Christian Kretzer ^a, §^, Blerina Shkodra ^b, c, §^, Paul Klemm ^b, c^, Paul M. Jordan ^a^, Daniel Schröder ^d^, Gizem Cinar ^b, c^, Antje Vollrath ^b, c^, Stephanie Schubert ^c, e^, Ivo Nischang ^b, c^, Stephanie Hoeppener ^b, c^, Steffi Stumpf ^b, c^, Erden Banoglu ^f^, Frederike Gladigau ^g, h, I^, Rossella Bilancia ^J^, Antonietta Rossi ^J^, Christian Eggeling ^c, d, g, k^, Ute Neugebauer ^c, g, h, I^, Ulrich S. Schubert ^b, c,^ *, Oliver Werz ^a, c,^ *

§ authors contributed equally

^a^Department of Pharmaceutical/Medicinal Chemistry, Institute of Pharmacy, Friedrich Schiller University Jena, Philosophenweg 14, 07743 Jena, Germany

^b^Laboratory of Organic and Macromolecular Chemistry (IOMC), Friedrich Schiller University Jena, Humboldtstraße 10, 07743 Jena, Germany

^c^Jena Center for Soft Matter (JCSM), Friedrich Schiller University Jena, Philosophenweg 7, 07743 Jena, Germany

^d^Institute for Applied Optics and Biophysics, Friedrich Schiller University Jena, Max-Wien Platz 1, 07743 Jena, Germany

^e^Department of Pharmaceutical Technology and Biopharmacy, Institute of Pharmacy, Friedrich Schiller University Jena, Lessingstraße 8, 07743 Jena, Germany

^f^Department of Pharmaceutical Chemistry, Faculty of Pharmacy, Gazi University, Etiler, 06330 Yenimahalle, Ankara, Turkey

^g^Leibniz Institute of Photonic Technology, Albert-Einstein-Straße 9, 07745 Jena, Germany

^h^Center for Sepsis Control and Care, Jena University Hospital, 07747 Jena, Germany

^i^Institute of Physical Chemistry and Abbe Center of Photonics, Helmholtzweg 4, 07743 Jena, Germany

^J^Department of Pharmacy, School of Medicine and Surgery, University of Naples Federico II, Via D. Montesano 49, I-80131 Naples, Italy.

^k^ MRC Human Immunology Unit, Weatherall Institute of Molecular Medicine, University of Oxford, Headley Way, OX39DS Oxford, UK


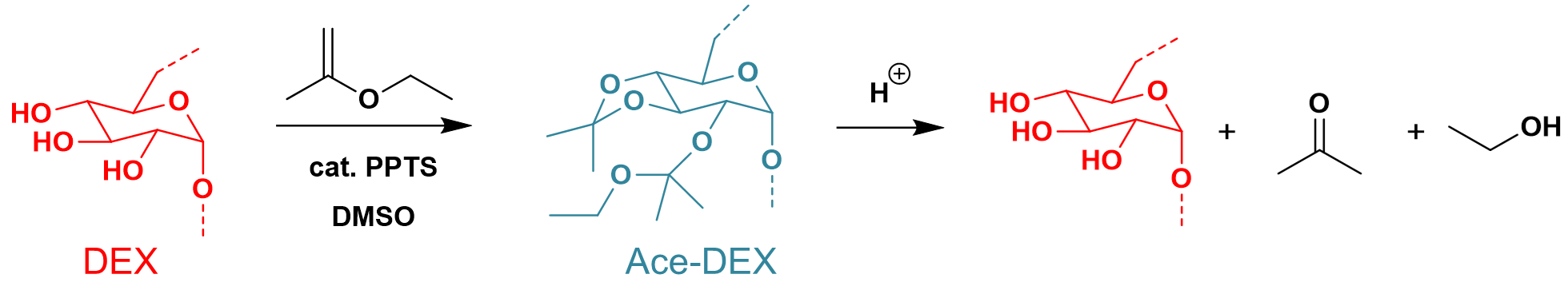


Figure S1. Synthesis of ethoxy acetalated dextran (Ace-DEX) and its degradation products upon hydrolysis.


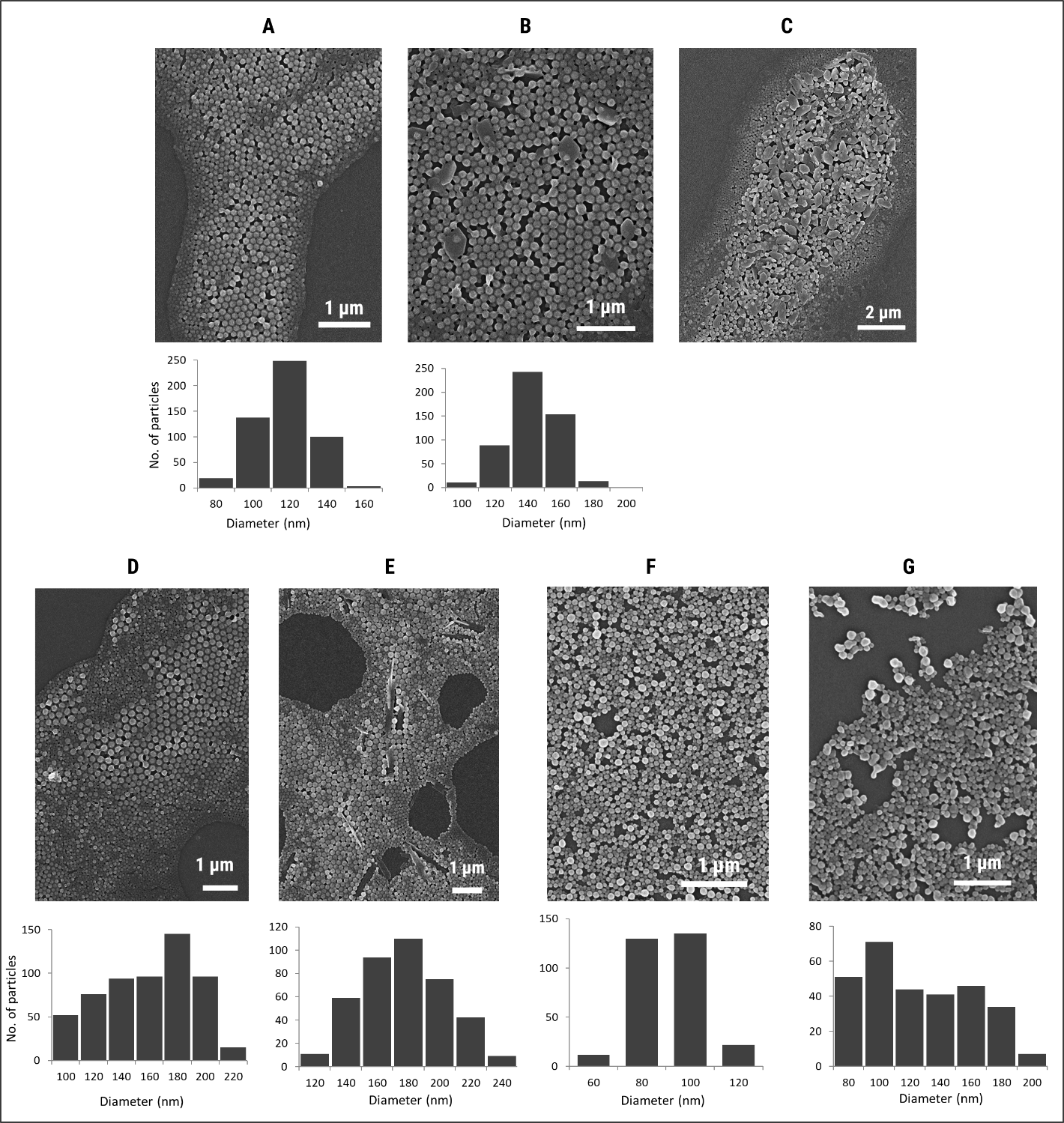


Figure S2. Scanning electron micrographs of the NPs: PLGA (A), PLGA[BRP-201] (B and C),
PLGA-Rho (D), PLGA-Rho[BRP-201] (E), Ace-DEX-Rho (F), Ace-DEX-Rho[BRP-201] (G). Histograms were generated from ImageJ measurements (n = 300 to 500).

##
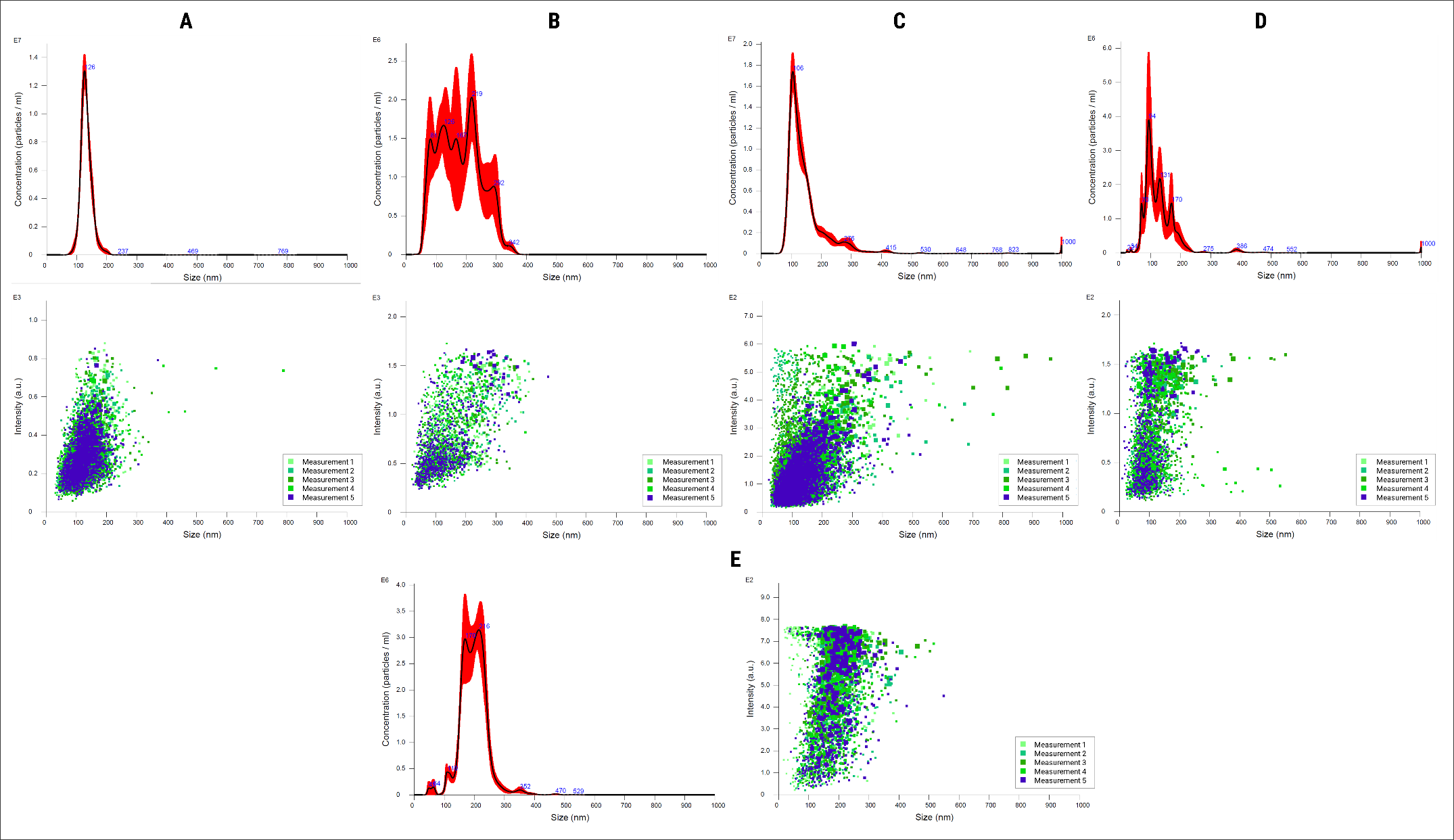
Figure S3. NTA measurements of the particle size (top row) and intensity distribution (bottom row): PLGA (A), PLGA[BRP-201] (B), Ace-DEX (C), Ace-DEX[BRP-201] (D), and BRP-201 precipitates (E).


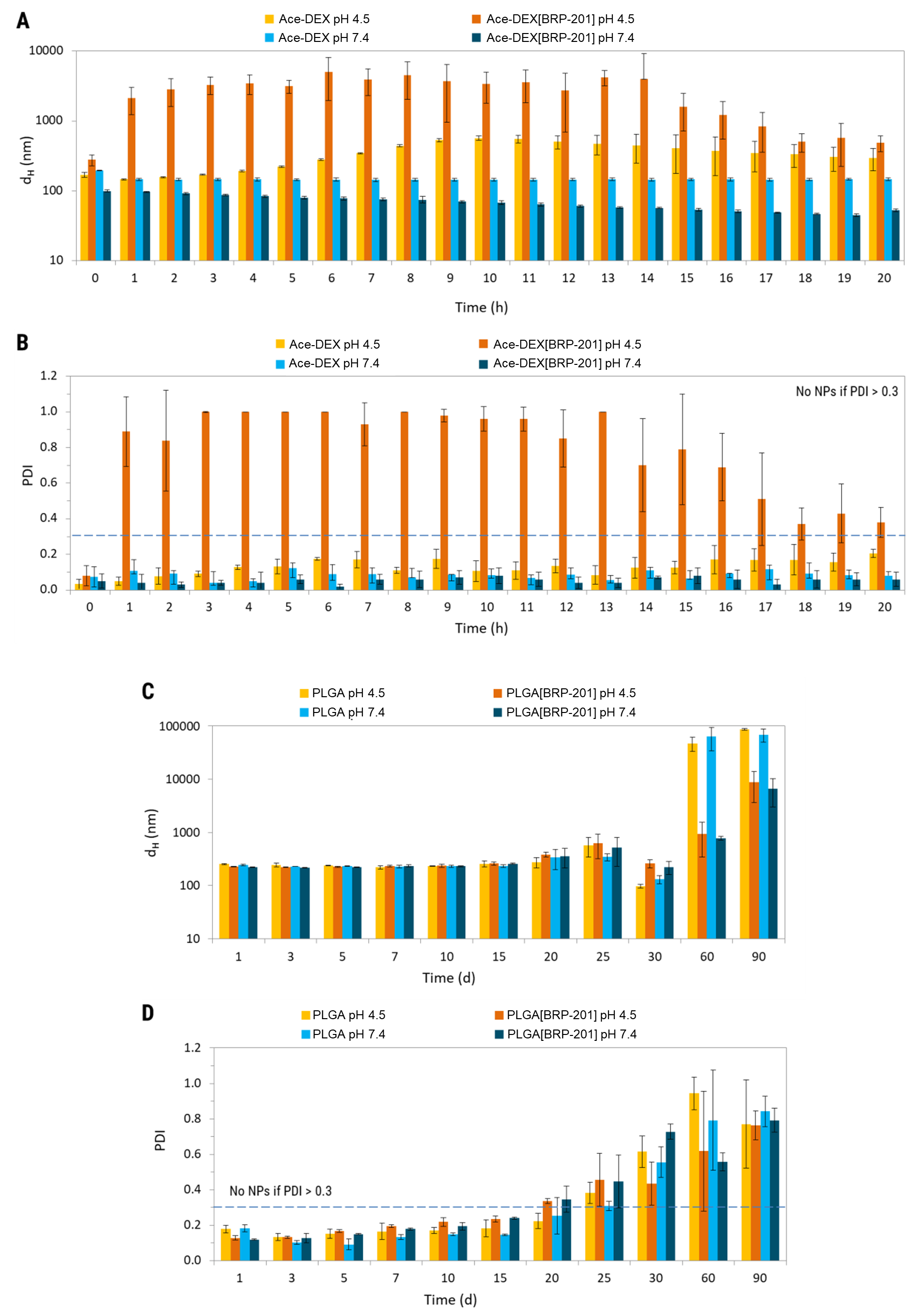


Figure S4. Size (A and C) and PDI (B and D) of the NPs over the 20 h measurements at 37 °C determined by DLS; NPs were incubated with 0.05 mM acetate buffer (pH 4.5) and 0.05 mM phosphate buffer (pH 7.4).


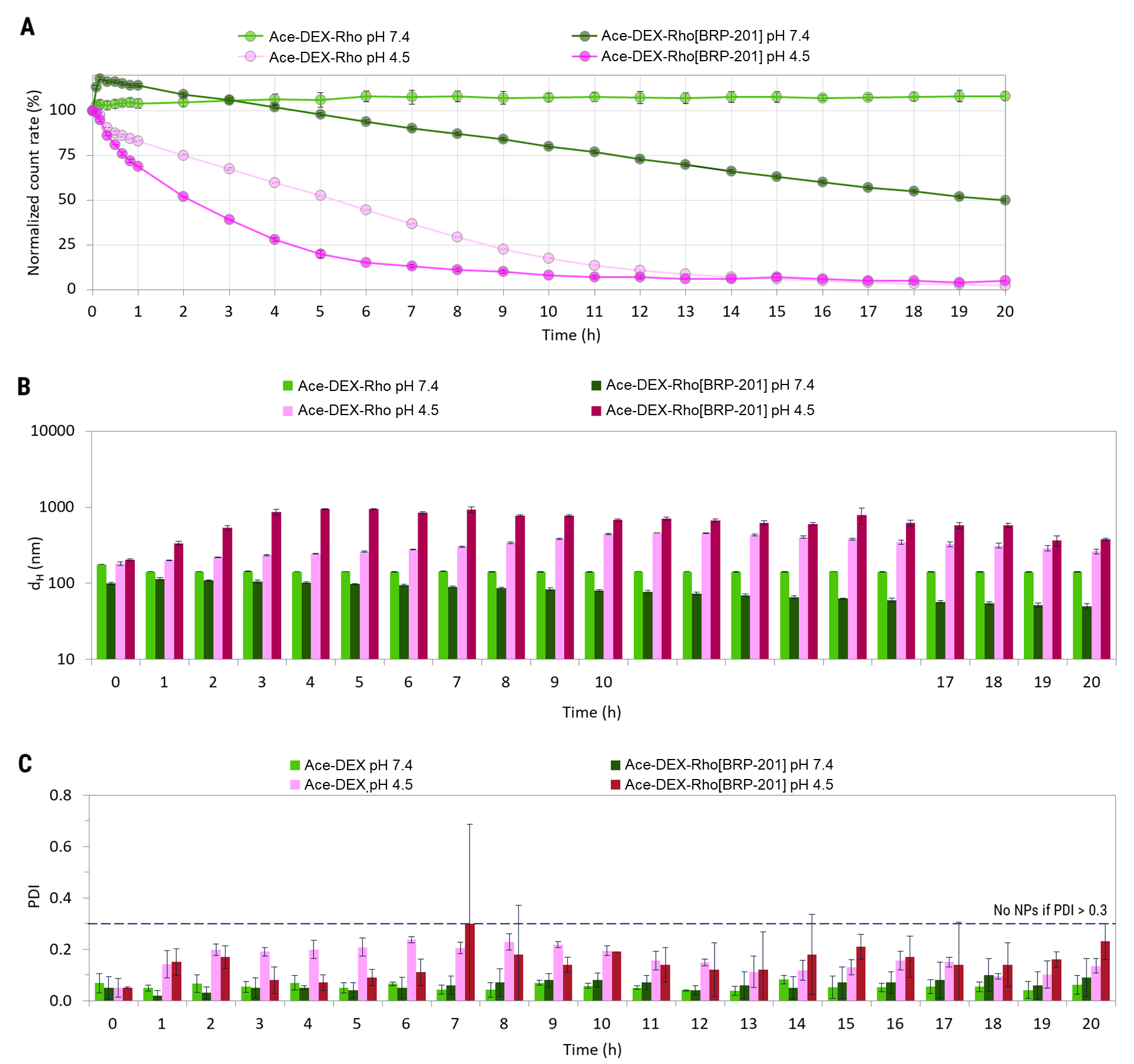


Figure S5. Degradation of Ace-DEX-Rho NPs at 37 °C incubated with 0.05 mM acetate buffer (pH 4.5) and 0.05 mM phosphate buffer (pH 7.4), as measured by DLS (n = 3) (A); the derived count rate on DLS was measured over predetermined time points, and plotted as normalized value against the derived count rate at timepoint 0 of incubation with buffer solution; concomitant size (B) and PDI (C) of the NPs over the 20 h measurements at 37 °C.


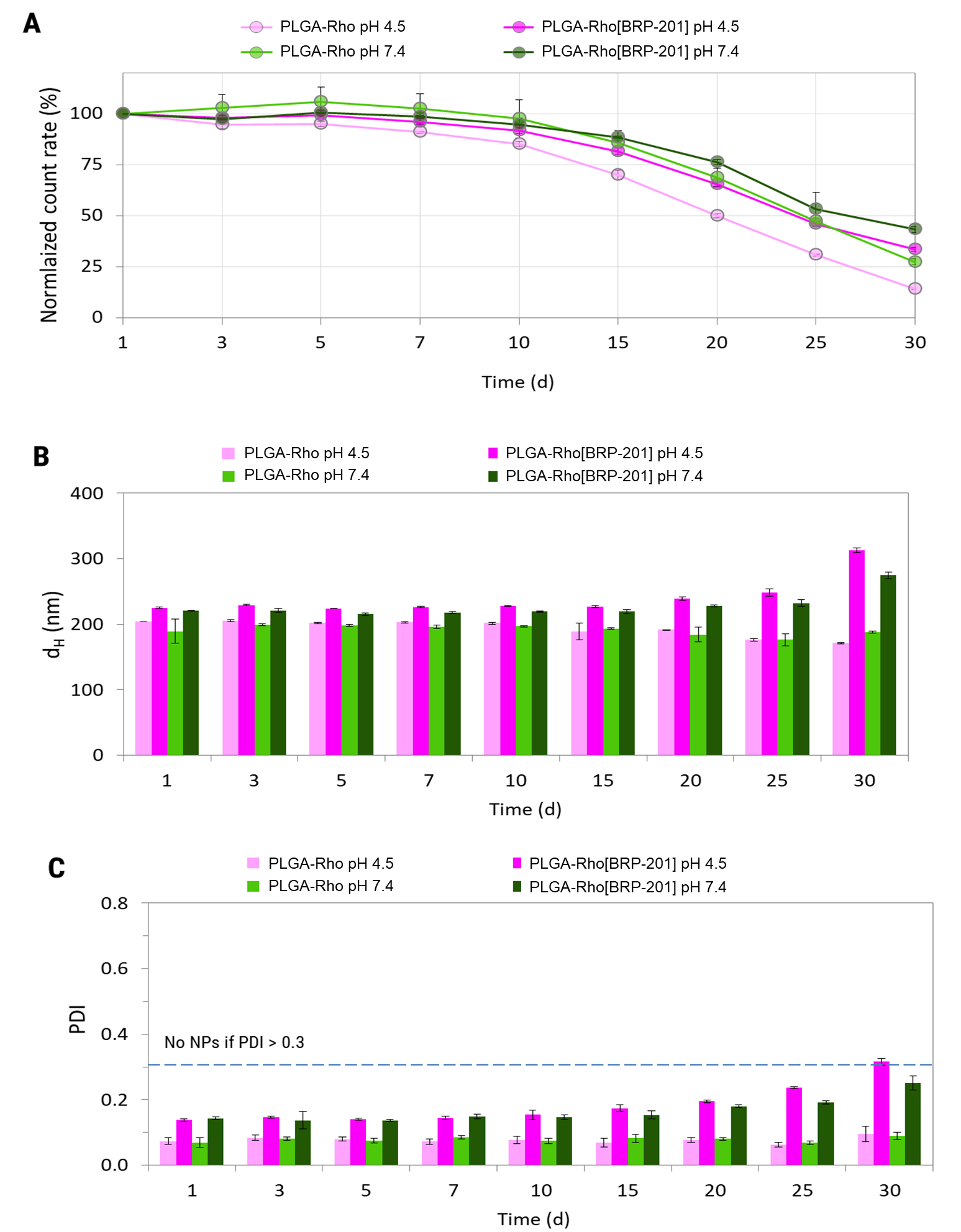


Figure S6. Degradation of Ace-DEX-Rho NPs at 37 °C incubated with 0.05 mM acetate buffer (pH 4.5) and 0.05 mM phosphate buffer (pH 7.4), as measured by DLS (n = 3) (A); the derived count rate on DLS was measured over predetermined time points, and plotted as normalized value against the derived count rate at timepoint 0 of incubation with buffer solution; concomitant size (B) and PDI (C) of the NPs over the 20 h measurements at 37 °C.


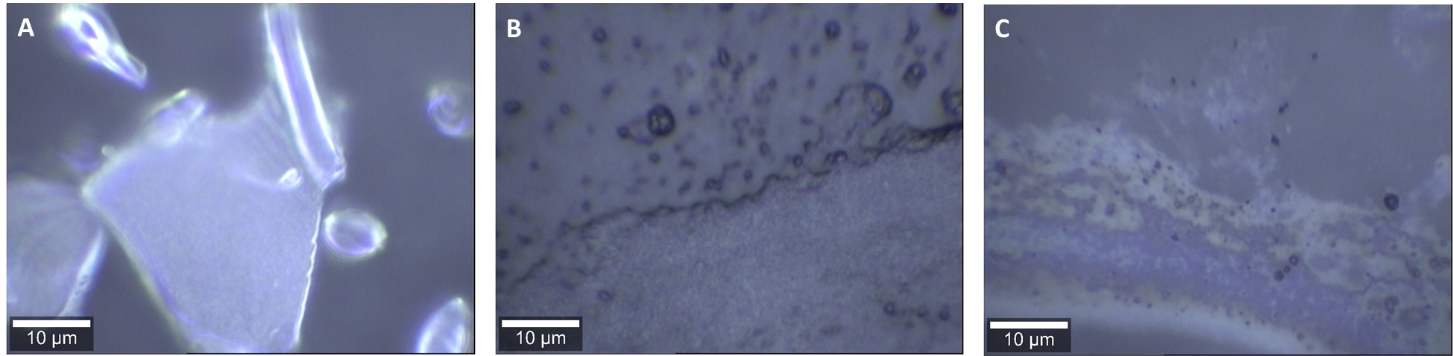


Figure S7. White light images of the drop coated sample of suspension of Ace-DEX[BRP-201]: (A) untreated Ace-DEX[BRP-201], (B) Ace-DEX[BRP-201] after 48h incubation at pH 4.5, (C) Ace-DEX[BRP-201] after 48h incubation at pH 7.4. It can be seen, that while initially large aggregations are present in the sample, after 48h incubation hardly any material is found on the slide. Raman spectra of the dried samples B and C are shown in Figure S8.


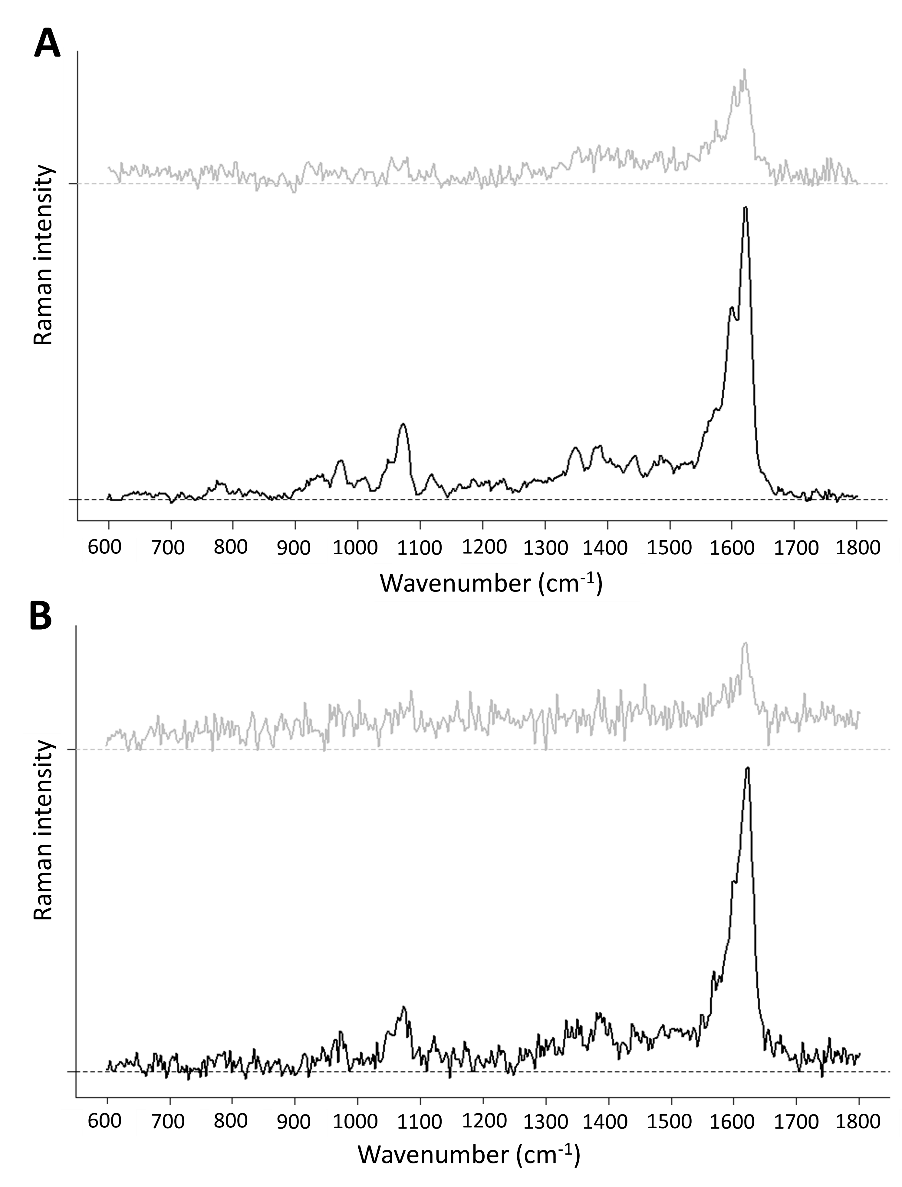


Figure S8. Spectra of the dried sample of Ace-DEX with BRP-201 after 48 h at pH 4.5 (A) and pH 7.4 (B). The upper (grey) spectra show the lowest intensity of the peak at 1620 cm^-1^ compared to the background, while the lower (black) spectra show the highest intensity. Peaks specific for Ace-DEX cannot be found in the spectra.


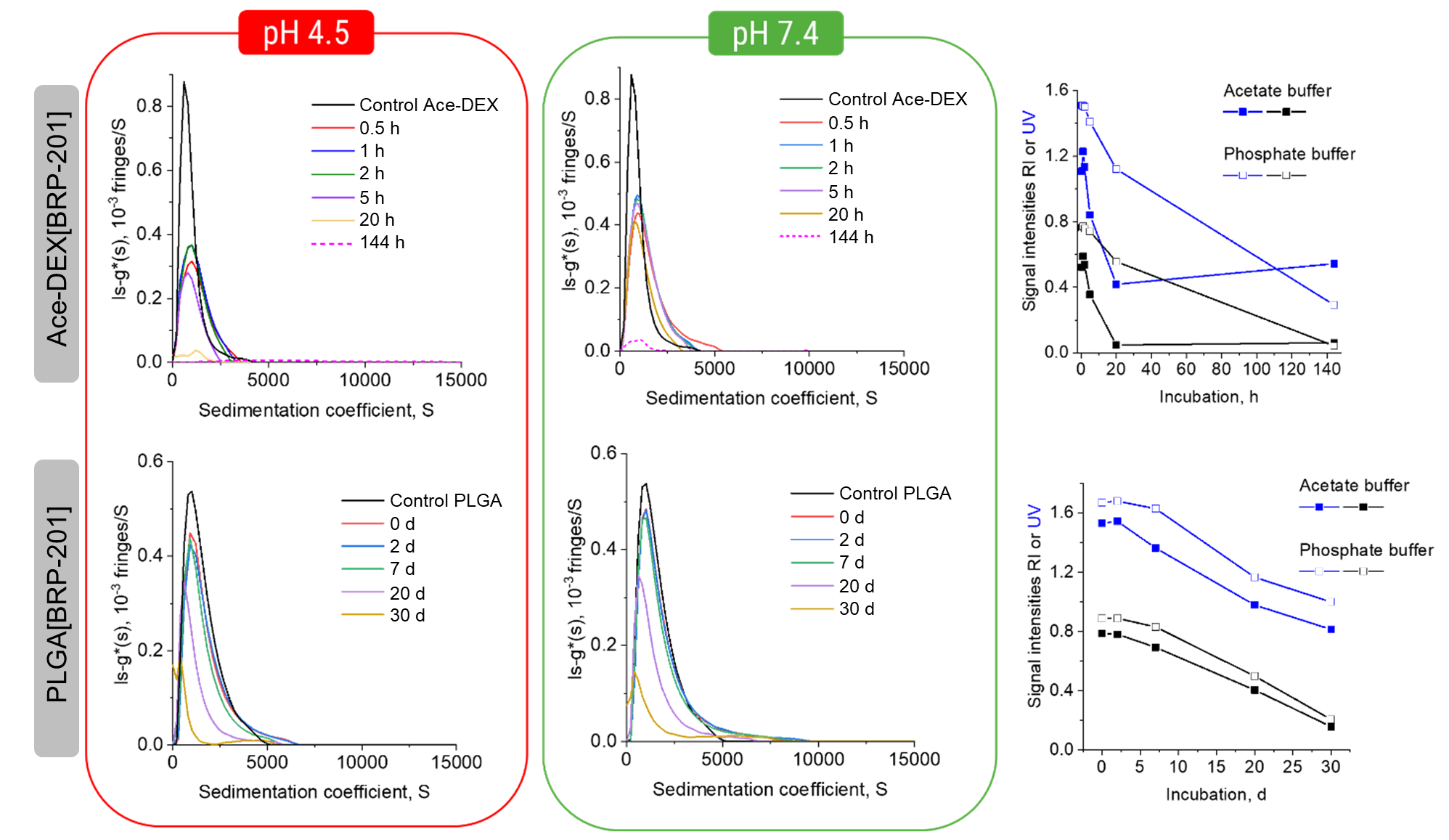


Figure S9. AUC investigation *via* refractive index (RI) detection of BRP-201-loaded NPs, incubated with buffers at 37 °C. Differential distribution of sedimentation coefficients, ls - g*(s), of Ace-DEX[BRP-201] NPs (top row) and PLGA[BRP-201] NPs (bottom row) incubated at 37 °C at different times and from experiments at a rotor speed of 1,500 rpm in acetate buffer (pH 4.5) (left) and phosphate buffer (pH 7.4) (middle). RI and UV (in terms of OD) signal received by integration of differential distributions of sedimentation coefficients, ls-g*(s), of particles against the incubation time at pH 4.5 and pH 7.4 (right). The control refers to NPs in water stored at 4 °C prior to the measurement.


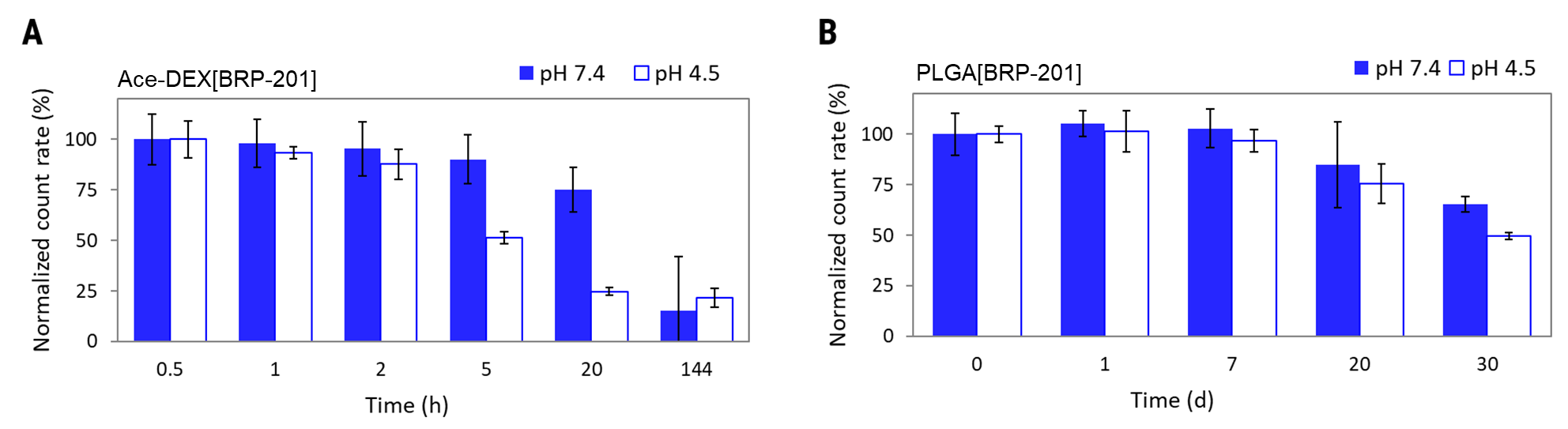


Figure S10. Degradation of NPs measured by DLS at the timepoints chosen for the degradation and/or the drug release measurements conducted on the AUC.

The RI signal intensity increased with longer incubation time at both pH values, probably due to the raise of the PVA amount in the supernatant as the NPs degraded [34] (Figure S12 and S13). For PLGA[BRP-201] NPs (Figure S13) the signal intensity of PVA in the supernatant was more pronounced for the NPs incubated at pH 4.5, even for the shortest incubation time, compared to pH 7.4. This may be a hint toward more pronounced degradation under acidic conditions.


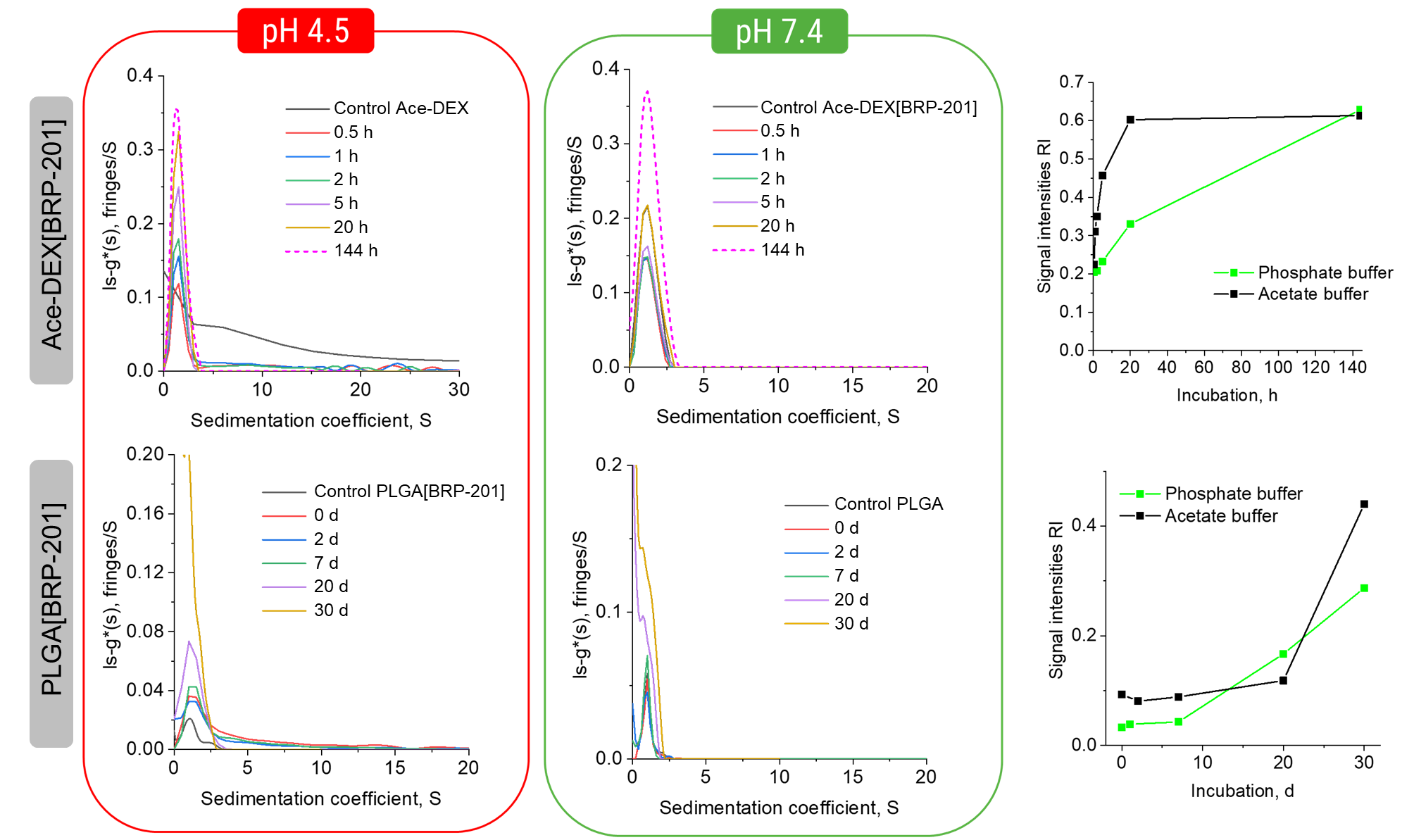


Figure S11. Differential distribution of sedimentation coefficients, ls - g*(s), of Ace-DEX[BRP-201] (top row) and PLGA[BRP-201] NPs (bottom row) incubated at 37 °C, at pH 4.5 (left) and pH 7.4 (middle) measured at a rotor speed of 42,000 rpm after an overall 32 h of centrifugation at lower speed of 1500 rpm in order to observe the remaining supernatant. RI signal intensities received by integration of differential distributions of sedimentation coefficients, ls-g*(s), of PVA in the supernatant against the incubation time (right).


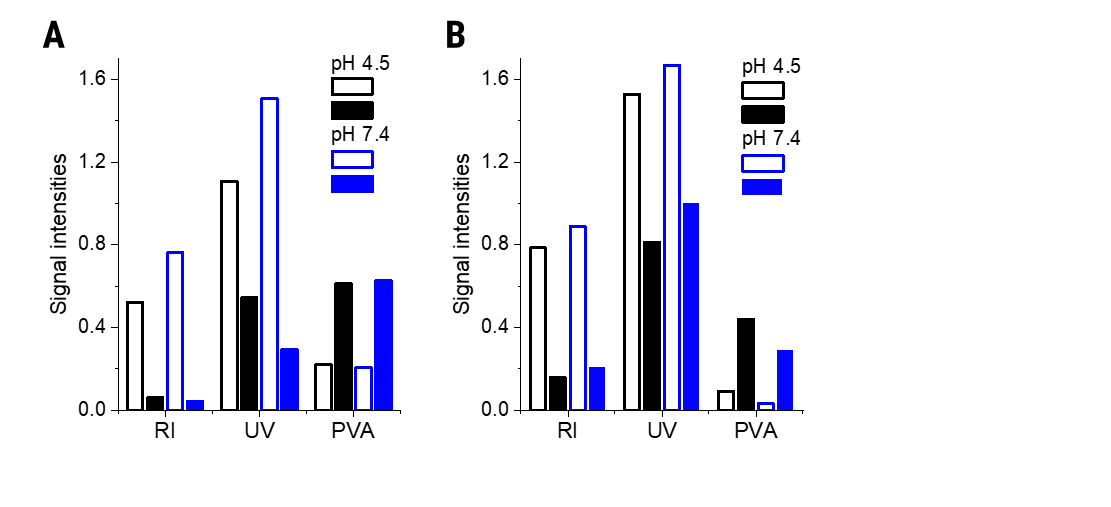


Figure S12. Signal intensities received by integration of differential distributions of sedimentation coefficients, ls-g*(s), representative of solution composition of Ace-DEX[BRP-201] NPs incubated for 0.5 h (empty bars) and of the NPs incubated for 144 h (filled bars) (A), and of PLGA[BRP-201] NPs incubated for 0 d (empty bars) and of the NPs incubated for 30 d (filled bars) (B).

Table S1. NTA measurement settings for individual NP-samples.

| **Formulation** | **C (µg mL^-1^)** | | **Screen gain** | **Camera level** | **Focus** | **Detection threshold** | **Particles/frame** |
| --- | --- | --- | --- | --- | --- | --- | --- |
| PLGA | 50 | 10 | | 8 | 26 | 4 | 27 |
| PLGA[BRP-201] | 50 | 11 | | 5 | 26 | 4 | 32 |
| Ace-DEX | 10 | 10 | | 11 | 19 | 7 | 71 |
| Ace-DEX[BRP-201] | 10 | 3 | | 15 | 16 | 29 | 70 |
| BRP-201 precipitates | 10 | 3 | | 8 | 14 | 14 | 78 |

Table S2. Overview of the size of the particles analyzed by different analytical methods.

| **Formulation** | **DLS intensity (nm)** | | **DLS number (nm)** | **NTA mean (nm)** | **NTA mode (nm)** | **SEM (nm)** |
| --- | --- | --- | --- | --- | --- | --- |
| PLGA | 165 ± 13 | 136 ± 9 | | 131 ± 19 | 123 ± 19 | 108 ± 15 |
| PLGA[BRP-201] | 185 ± 5 | 139 ± 7 | | 179 ± 70 | 218 ± 70 | 133 ± 15 |
| Ace-DEX | 180 ± 13 | 104 ± 6 | | 148 ± 76 | 105 ± 76 | 70 ± 10 |
| Ace-DEX[BRP-201] | 111 ± 13 | 79 ± 16 | | 129 ± 66 | 94 ± 66 | 76 ± 13 |
| BRP-201 precipitates | 317 ± 44 | 252 ± 38 | | 198 ± 45 | 215 ± 45 | / |

Table S3. Summary of the physicochemical properties of the pooled NP-formulation loaded with 10% (w/w) BRP-201.

| **Pooled NPs  with 10% BRP-201** | **Purified NP suspension** | | | **NP suspension + 0.9% NaCl** | | | | **Yield** | | **PVA** | **EE** | **LC** |  |
| --- | --- | --- | --- | --- | --- | --- | --- | --- | --- | --- | --- | --- | --- |
|  | **d_H_ (nm)** | **PDI** | **ζ (mV)** | | **d_H_ (nm)** | **PDI** | **ζ (mV)** | | **(%)** | **(%, w/w)** | **(%)** | **(%, w/w)** | |
| Ace-DEX | 158 | 0.10 | -8 | | 152 | 0.03 | -2 | | 58 | 8.3±0.2 | / | / | |
| Ace-DEX[BRP-201] | 237 | 0.24 | -16 | | 231 | 0.23 | -1 | | 66 | 5.5±0.3 | 108±11 | 98±12 | |

Table S4. Particle size distribution of the NP suspensions for *in vivo* evaluation as measured *via* multi-angle light scattering (MADLS) with Zetasizer Ultra.

| **Formulations  for *i.v.* injection** | **MADLS** | **Purified NP suspension** | | | | | **NP suspension + 0.9% NaCl** | | | | |
| --- | --- | --- | --- | --- | --- | --- | --- | --- | --- | --- | --- |
|  | **Angle** | **Peak 1**  **d_H_ (nm)** | **Peak 1 (%)** | **Peak 2 d_H_ (nm)** | **Peak 2 (%)** | **Peak 1**  **d_H_ (nm)** | | **Peak 1 (%)** | **Peak 2 d_H_ (nm)** | **Peak 2  (%)** |  |
| Ace-DEX | 173° | 167 | 100 | 0 | 0 | 162 | | 100 | / | 0 |  |
|  | 90° | 182 | 100 | / | 0 | 166 | | 100 | / | 0 |  |
|  | 13° | 138 | 95 | 2 | 5 | 162 | | 100 | / | 0 |  |
| Ace-DEX[BRP-201] | 173° | 243 | 100 | / | 0 | 268 | | 100 | / | 0 |  |
|  | 90° | 320 | 89 | 57 | 11 | 338 | | 90 | 61 | 11 |  |
|  | 13° | 150 | 68 | 648 | 32 | 157 | | 79 | 3344 | 21 |  |

Table S5. Particle size distribution of the NP suspensions for *in vivo* evaluation measured *via* multi-angle light scattering (MADLS) with Zetasizer Ultra after 28 days storage at 4 °C.

| **Formulations  for *i.v.* injection** | **MADLSAngle** | **Peak 1 d_H_ (nm)** | | **Peak 1  (%)** | **Peak 2 d_H_ (nm)** | **Peak 2 (%)** |
| --- | --- | --- | --- | --- | --- | --- |
| Ace-DEX | 173° | | 158 | 100 | / | 0 |
|  | 90° | | 165 | 100 | / | 0 |
|  | 13° | | 134 | 96 | 5161 | 3 |
| Ace-DEX[BRP-201] | 173° | | 638 | 95 | 57 | 5 |
|  | 90° | | 1064 | 57 | 391 | 43 |
|  | 13° | | 287 | 100 | 0 | 0 |

Figure S13. SEM micrographs of Ace-DEX NPs (A and B) and Ace-DEX[BRP-201] NPs (C and D) with 0.9% NaCl.
